# Two calcium pyrene phospho­nate frameworks with contrasting dimensionality and porosity

**DOI:** 10.1107/S2052252526009875

**Published:** 2026-11-06

**Authors:** Pablo Salcedo-Abraira, Rubén Serrano-Nieto, Sergio Carrasco, Catalina Biglione, Inhar Imaz, Thomas Devic, Daniel Maspoch, Patricia Horcajada

**Affiliations:** a Department of Inorganic Chemistry University of Granada Avda. Fuente nueva s/n 18071Granada Spain; b Nantes Université, CNRS Institut des Matériaux de Nantes Jean Rouxel, IMN 44000Nantes France; c IMDEA Energy Institute Advanced Porous Materials Unit (APMU) Avda. Ramón de la Sagra 3 28935 Móstoles Madrid Spain; d Catalan Institute of Nanoscience and Nanotechnology (ICN2) CSIC and The Barcelona Institute for Science and Technology Campus UAB, Bellaterra 08193Barcelona Spain; e ICREA Pg. Lluís Companys 23 08010Barcelona Spain; Stellenbosch University, South Africa

**Keywords:** metal–organic frameworks, MOFs, calcium phospho­nate, coordination polymers, microporous materials

## Abstract

Two new calcium phospho­nate metal–organic frameworks (P-MOFs), IEF-33 and IEF-34, have been solvothermally synthesized from a pyrene tetra­phospho­nate ligand and structurally characterized by single-crystal X-ray diffraction. The two compounds arise from different degrees of ligand hydrolysis and exhibit distinct architectures: IEF-33 forms a 2D layered framework interacting through hydrogen bonds with interlayer water-filled channels, whereas IEF-34 adopts a 3D porous network with 1D channels. Gas sorption measurements demonstrate the permanent porosity of IEF-34, which exhibits a BET surface area of 491 m^2^ g^−1^ and a CO_2_ uptake of 1.6 mmol g^−1^ at 273 K. Both materials show good chemical stability in aqueous and organic media, and moderate thermal stability. In addition, IEF-33 and IEF-34 show strong visible-light absorption with optical band gaps of 2.51 and 2.62 eV, respectively.

## Abstract 2

The synthesis and structures of two novel porous calcium-based phospho­nate MOFs are reported, and their chemical stability and light absorption properties are investigated.

## 1. Introduction

Metal–organic frameworks (MOFs) have emerged over the last few decades as one of the most interesting and versatile classes of crystalline porous materials. Built from inorganic nodes interconnected by organic linkers, these materials present an extraordinary structural and functional diversity combined with high modularity, resulting in an exponential growth of the number of reported structures of MOFs [there are now >110 000 entries in the Cambridge Structural Database (CSD); see https://support.ccdc.cam.ac.uk/support/solutions/articles/103000306242-how-many-mofs-are-there-in-the-csd-] as well as of their potential applications in diverse fields such as fluid storage and separation, catalysis, sensing, medicine and energy (Furukawa *et al.*, 2013 ▸; Li *et al.*, 2009 ▸; Kumar *et al.*, 2015 ▸; Horcajada *et al.*, 2012 ▸; Xia *et al.*, 2015 ▸).

Despite this remarkable structural diversity, the vast majority of reported MOFs rely on carboxyl­ate-based linkers. While these materials often exhibit excellent porosity and functionality, their chemical and hydro­thermal stability remain significant challenges (An *et al.*, 2024 ▸). In this context, phospho­nate-based MOFs (P-MOFs) have attracted researchers’ attention because of the stronger metal–oxygen bonds associated with the phospho­nate group, which generally confer superior thermal, chemical and hydrolytic robustness than their carboxyl­ate counterparts (Gagnon *et al.*, 2012 ▸; Shearan *et al.*, 2019 ▸; Mysore Ramesha & Meynen, 2020 ▸). However, because of this coordination versatility and the high charge of the phospho­nate groups, these types of ligands frequently promote the formation of dense inorganic sublattices with complex connectivity and low crystallinity, complicating their structural elucidation. As a result, despite their attractive stability, the number of structurally resolved and permanently porous P-MOFs remains relatively limited (Zheng *et al.*, 2024 ▸).

In parallel, the growing dependence on critical raw materials and the increasing geopolitical uncertainties affecting their availability have stimulated the search for MOFs based on earth-abundant sustainable elements (see https://single-market-economy.ec.europa.eu/sectors/raw-materials/areas-specific-interest/critical-raw-materials_en). In this con­text, calcium appears particularly attractive owing to its abundant nature (it is the fifth most abundant element in the Earth’s crust, at 3.6–4.1%), low toxicity, low cost and excellent environmental compatibility. Nevertheless, calcium-based MOFs remain severely under-represented compared with their transition-metal counterparts. This scarcity can be largely attributed to the large ionic radius and high coordination versatility of Ca^2+^, which typically favor dense crystal packing and structural instability upon activation, thereby hindering the development of permanently porous materials (Fromm, 2008 ▸; Xian *et al.*, 2021 ▸).

Such structural challenges are further exacerbated in calcium phospho­nate frameworks, where the strong metal–oxygen interactions and multitopic nature of phospho­nate ligands often favor the crystallization of compact, non-porous architectures rather than the intended open-framework materials. To date, fewer than 160 calcium phospho­nate structures have been reported in the CSD, and fewer than 60 correspond to coordination polymers exclusively constructed from Ca^2+^ cations and phospho­nate ligands (*i.e.* without additional coordinating functionalities). Among these, only around 20 structures satisfy the IUPAC definition of a MOF (*i.e.* structures containing potential voids) (Batten *et al.*, 2013 ▸). From these structures, only two studies have explored their adsorption properties through H_2_O and NH_3_ sorption measurements (Colodrero *et al.*, 2009 ▸) or by N_2_ and CO_2_ uptake (Glavinović *et al.*, 2022 ▸). This scarcity of adsorption studies highlights a significant knowledge gap regarding the true porous potential of this family of materials.

Within this context, the use of rigid, multitopic phospho­nate ligands emerges as a promising strategy for obtaining porous structures (Taddei *et al.*, 2016 ▸; Firmino *et al.*, 2018 ▸). Pyrene-based tetra­phospho­nates are particularly interesting because of their aromatic core, high rigidity and intrinsic optoelectronic properties, which have already demonstrated good performance when incorporated into hybrid materials (Salcedo-Abraira, Serrano-Nieto *et al.*, 2023 ▸; Serrano-Nieto *et al.*, 2025 ▸; Firmino *et al.*, 2020 ▸; Salcedo-Abraira, Biglione *et al.*, 2023 ▸). Nevertheless, only one calcium pyrene tetra­phospho­nate structure has been reported so far. This material corresponds to a non-porous coordination network (Serrano-Nieto *et al.*, 2025 ▸), leaving the exploration of porosity and adsorption behavior in this system essentially unexplored.

In this work, we report the synthesis and full structural characterization of two novel calcium-based P-MOFs (labeled IEF-33 and IEF-34 from IMDEA Energy Framework) containing the pyrene tetra­phospho­nate ligand. The thermal and structural stability, and textural and optoelectronic properties of both materials were also investigated. IEF-33 is built from the fully hydrolyzed and partially deprotonated version of the ligand [Py(PO_3_Et_2_)_4_], *i.e.* [Py(PO_3_H)_4_]^4−^, resulting in a two-dimensional coordination network connectivity in which Ca^2+^ centers are coordinated by both phospho­nate groups and water molecules, with adjacent layers interconnected through hydrogen bonds. In contrast, IEF-34 contains the partially hydrolyzed and deprotonated ligand [Py(PO_3_Et)_2_(PO_3_HEt)_2_]^2−^ with the Ca atoms totally surrounded by the phospho­nate groups, leading to a three-dimensional network with void channels (with a specific surface BET area of 490 m^2^ g^−1^) that are accessible to both N_2_ and CO_2_ gases. Both materials combined a good structural stability in organic media, relative stability in aqueous media and enhanced light absorption compared with the pristine ligand.

## 2. Materials and methods

All the chemicals were used as received without further pur­ification: Ca(NO_3_)_2_·4H_2_O (99%, Alfa Aesar, 236.15 g mol^−1^), pyrene (98%, Sigma Aldrich, 202.25 g mol^−1^), bromine (≥95.5%, Sigma Aldrich, 159.81 g mol^−1^), nitro­benzene (99%, Thermo Scientific), P(OEt)_3_ (98%, Sigma Aldrich, 166.16 g mol^−1^), [Pd(PPh_3_)_4_] (99%, Sigma Aldrich, 1155.56 g mol^−1^), EtOH (≥99.8%, ACS reagent), MeOH (≥99.8%, ACS reagent), *n*-hexane (technical grade, VWR Chemicals) and HCl (37%, ACS reagent). Py(PO_3_Et_2_)_4_ was synthesized using a previously reported procedure (see the supporting information for further information) (Salcedo-Abraira, Serrano-Nieto *et al.*, 2023 ▸).

### 2.1. Synthesis

#### 2.1.1. Single-crystal synthesis of IEF-33, {[Ca_2_(C_16_H_10_P_4_O_12_)­(H_2_O)_4_]·3H_2_O}*_n_* (molecular weight 724.39 g mol^−1^)

In a 2.5 ml Teflon-lined reactor, 10.63 mg (0.045 mmol) of Ca(NO_3_)_2_·4H_2_O and 16.79 mg (0.0225 mmol) of Py(PO_3_Et_2_)_4_ were dissolved in 0.5 ml of MeOH and 1 ml of distilled water, then the reactor was closed. The reactor was heated in an oven to 120 °C over 6 h, maintained at this temperature for 48 h and finally cooled down to 30 °C over 6 h. The resulting brown crystals were recovered by filtration and washed with distilled water and methanol, and were dried at room temperature (RT).

#### 2.1.2. Scaled-up synthesis of IEF-33

In a 25 ml Teflon-lined reactor, 106.3 mg (0.45 mmol) of Ca(NO_3_)_2_·4H_2_O and 167.9 mg (0.225 mmol) of Py(PO_3_Et_2_)_4_ were dissolved in 5 ml of MeOH and 10 ml of distilled water, then the reactor was closed. The reactor was heated in an oven at 120 °C for 87 h. The resulting brown crystals were recovered by filtration and washed with distilled water and methanol, and were dried at RT. Solid recovered: 90 mg (70 ± 5% yield based on the linker).

#### 2.1.3. Single-crystal synthesis of IEF-34, [Ca(C_20_H_20_P_4_O_12_)]*_n_* (molecular weight 616.32 g mol^−1^)

In a 2.5 ml Teflon-lined reactor, 10.63 mg (0.045 mmol) of Ca(NO_3_)_2_·4H_2_O and 16.79 mg (0.0225 mmol) of Py(PO_3_Et_2_)_4_ were dissolved in 1 ml of EtOH and 0.5 ml of distilled water. Then 10 µl of 5 *M* HCl were added and the reactor was closed. The reactor was heated in an oven to 120 °C over 8 h, maintained at this temperature for 48 h and finally cooled down to 30 °C over 8 h. The resulting yellow crystals were recovered by filtration and washed with distilled water and ethanol, and were dried at 100 °C for 12 h.

#### 2.1.4. Scaled-up synthesis of IEF-34

In a 25 ml Teflon-lined reactor, 98.52 mg (0.42 mmol) of Ca(NO_3_)_2_·4H_2_O and 155.84 mg (0.21 mmol) of Py(PO_3_Et_2_)_4_ were dissolved in 9.2 ml of EtOH and 4.6 ml of distilled water. Then 100 µl of 5 *M* HCl were added and the reactor was closed. The reactor was heated in an oven to 130 °C over 8 h, maintained at this temperature for 48 h and finally cooled down to 30 °C over 8 h. The resulting yellow crystals were recovered by filtration and washed with distilled water and ethanol, and were dried at 100 °C for 12 h. Solid recovered: 77 ± 4 mg (60 ± 3% yield based on the linker).

### 2.2. Crystallographic studies

Suitable single crystals of Py(PO_3_Et_2_)_4_, IEF-33 and IEF-34 were mounted on a MiTeGen polymer loop and covered with oil. Data were collected at room temperature using a Bruker Venture and a Rigaku XtaLAB Synergy diffractometer with Mo *K*α radiation (λ = 0.71073 Å) for Py(PO_3_Et_2_)_4_ and IEF-34, respectively. Data for IEF-33 were collected at the ALBA synchrotron (Spain) on the XALOC beamline at 100 K using a single φ-scan data acquisition strategy (Juanhuix *et al.*, 2014 ▸). The data for Py(PO_3_Et_2_)_4_ were reduced using *APEX3* and an absorption correction was applied using *SADABS*. For IEF-33, data indexing, integration and scaling were performed with the *XDS* program (Kabsch, 2010 ▸) and an absorption correction was not applied. Data reduction for IEF-34 was performed with the *CrysAlisPro* suite and a semi-empirical absorption correction (*ABSPACK*) was applied. The structures were solved using *SHELXT* (Sheldrick, 2015*b* ▸) and refined with *SHELXL* (Sheldrick, 2015*a* ▸) integrated within the *Olex2* graphical interface (Dolomanov *et al.*, 2009 ▸). The volumes of the solvent-accessible voids were calculated using a 1.2 Å probe. All non-H atoms were refined anisotropically. Hydrogen atoms were placed in calculated positions and refined with idealized geometries.

Powder X-ray diffraction (PXRD) data used for crystallographic studies were collected on a BRUKER D8 DISCOVER equipped with a microfocus Cu *K*α radiation source (λ = 1.54059 Å) over a 2θ range of 4–50° with a 0.02° step size and 40 s of acquisition time per step.

### 2.3. Instrumentation

Routine PXRD data were collected using an Empyrean Panalytical diffractometer (Cu *K*α, λ = 1.5406 Å) equipped with a PIXcel3D detector, operating at 45 kV and 40 mA from 3 to 35° 2θ with a step size of 0.013° and a scan rate of 0.1° s^−1^. Scanning electron microscopy (SEM) micrographs were collected on a Hitachi TM-1000 microscope operating at 15 kV. Fourier-transform infrared (FTIR) spectra were collected in the 4000 to 400 cm^−1^ range using a Thermo Nicolet 6700 FTIR spectrometer with an ATR accessory instrument (Thermo Scientific, USA). Elemental analyses were carried out in a Flash 2000 analyzer from Thermo Scientific. Inductively coupled plasma optical emission spectroscopy (ICP-OES) analyses were carried out using a 2300 DV spectrometer from Perkin Elmer. TGA was performed using an SDT650 thermobalance (TA instruments) over the 25–1000 °C range at 5 °C min^−1^ and under an air flow of 100 ml min^−1^. Variable-temperature (VT) PXRD data were acquired on a D8 Advance Bruker AXS θ–2θ diffractometer (Cu *K*α radiation, λ = 1.54060 Å) equipped with a LYNXEYE XE detector, operating at 40 kV and 40 mA, and an Anton Paar XRK 900 high-temperature chamber. Both nitro­gen (at 77 K) and CO_2_ (at both 273 K and 293 K) sorption isotherms were collected using a 3Flex instrument from Micromeritics. Prior to the gas sorption experiments, samples were degassed at 120 °C for 6 h under secondary vacuum. Diffuse reflectance UV–Vis measurements were recorded using a Perkin Elmer spectrometer (Lambda 19) equipped with an integrating sphere and a praying mantis accessory.

## 3. Results and discussion

### 3.1. Synthesis and structural elucidation

The reactivity of Ca^2+^ cations with the lab-made pyrene octa­ethyl tetra­phospho­nate ligand was investigated using the solvothermal high-throughput method (Stock, 2010 ▸). Upon synthetic optimization, two novel calcium phospho­nate phases were isolated, labeled as IEF-33 and IEF-34. The generation of one or the other phase was mostly determined by the mixture of solvents employed: IEF-33 required the use of an MeOH/H_2_O mixture in a 1:2 (*v*/*v*) ratio, while IEF-34 was obtained using an EtOH/H_2_O 2:1 (*v*/*v*) mixture with the addition of 1 equivalent of HCl per metal. Interestingly, the IEF-33 structure contained the totally hydrolyzed ligand, whereas in IEF-34 half of the phospho­nate groups in the ligand were in their esterified version (where HCl is used during the synthesis). Compared with the synthetic conditions previously reported for the dense calcium pyrene tetra­phospho­nate framework IEF-22(Ca) (Serrano-Nieto *et al.*, 2025 ▸), the preparation of the more open and porous IEF-33 and IEF-34 relies on significantly milder reaction conditions and a more straightforward synthetic approach. Specifically, both MOFs were obtained at lower temperatures (120–130 versus 150 °C), using a lower metal concentration (0.03 versus 0.1 *M*) and employing the esterified version of the ligand (Table 1 ▸, Tables S1 and S2, and Fig. S1) instead of its acidic counterpart. The latter was employed for IEF-22, requiring an additional synthetic step during ligand preparation.

**Figure 1:**
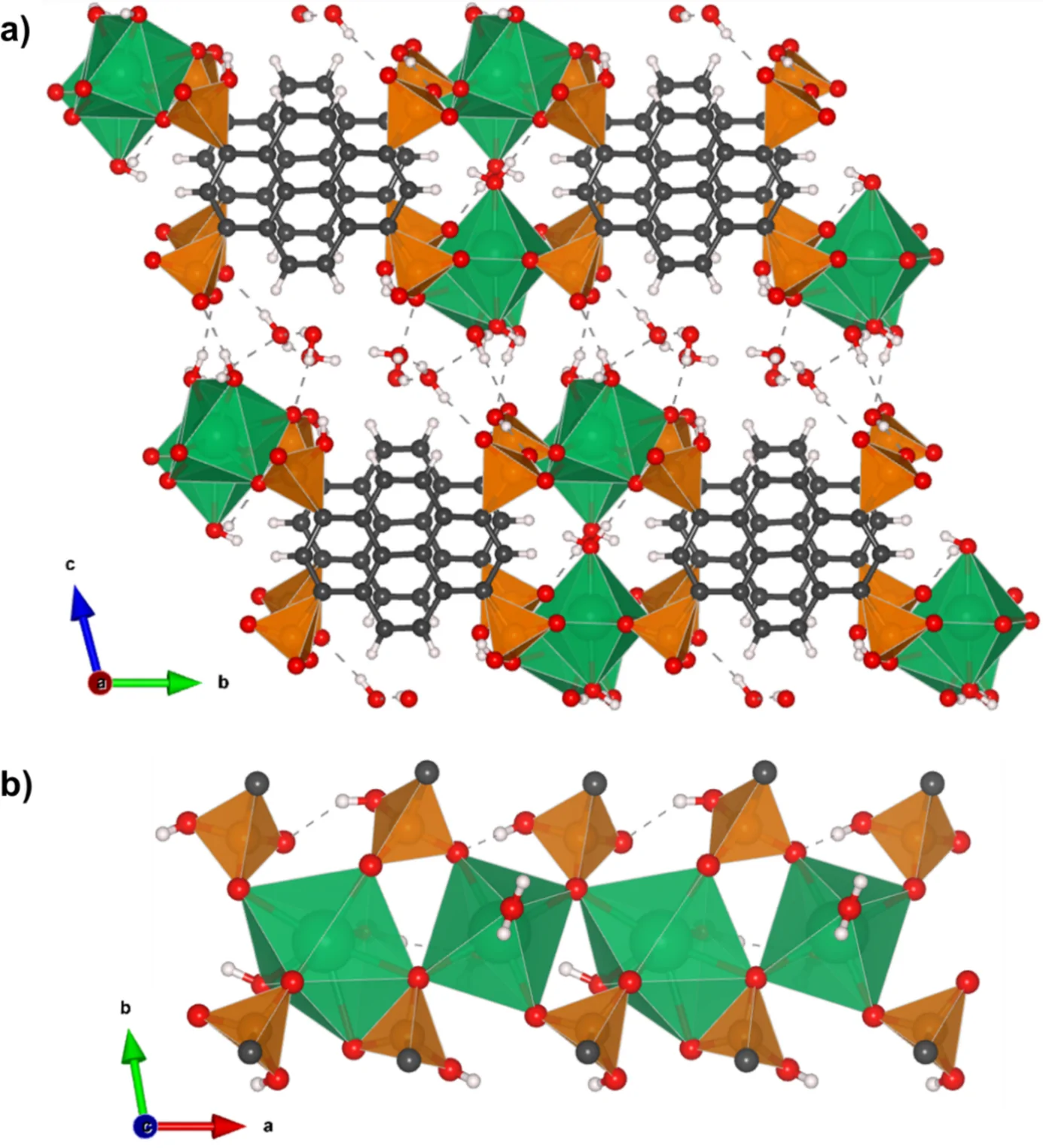
IEF-33 crystal structure viewed along the [100] direction (*a*) and the IEF-33 SBU viewed along the [001] direction (*b*). Calcium, phospho­rus, oxygen, carbon and hydrogen atoms are represented in green, orange, red, black and white, respectively.

IEF-33 was obtained as small brown needle-shaped crystals (∼75 µm long, Fig. S2), hindering structural determination by conventional SCXRD. Therefore, synchrotron SCXRD was employed, revealing that IEF-33, formulated as {[Ca_2_(C_16_H_10_P_4_O_12_)(H_2_O)_4_]·3H_2_O}*_n_* or {[Ca_2_(Py(PO_3_)_4_)­(H_2_O)_4_]·3H_2_O}*_n_*, crystallizes in the triclinic space group *P*1 (No. 2) with unit-cell parameters *a* = 8.4993 (3) Å, *b* = 12.5656 (9) Å, *c* = 12.5832 (1) Å, α = 103.252 (7)°, β = 98.700 (5)°, γ = 97.446 (4)°, *V* = 1274.05 (2) Å^3^ and *Z* = 2 (Table 1 ▸). The IEF-33 structure is built from [Ca_2_(Py(PO_3_)_4_)­(H_2_O)_4_]_*n*_ layers along the (001) plane, interacting through hydrogen bonds [Fig. 1 ▸(*a*)]. The asymmetric unit contains one fully hydrolyzed pyrene tetra­phospho­nate ligand coordinating two crystallographically independent Ca^2+^ cations (Ca1 and Ca2), each one with two coordinated water molecules, and three crystallization water molecules. The secondary building unit (SBU) consists of [Ca_2_(PO_3_H)_4_­(H_2_O)_4_]*_n_* chains running along the [100] direction [Fig. 1 ▸(*b*)]. These chains contain the Ca1 and Ca2 cations alternating in two different coordination spheres. Ca1 is found in a highly distorted [CaO_7_] pentagonal bipyramid environment with *d*(Ca1—O) distances between 2.362 (1) and 2.761 (1) Å [Tables S4 and S5, and Fig. S3(*a*)] and in a distorted [CaO_6_] octahedral environment with *d*(Ca2—O) distances between 2.279 (1) and 2.415 (1) Å [Tables S4 and S5, and Fig. S3(*b*)]. The Ca1 coordination sphere contains the P2 phospho­nate group of one ligand, which is acting as a chelating ligand (through O5 and O6), the P3 and P4 phospho­nate groups from different ligands acting as monodentate ligands (by O1, O8 and O11, respectively) and two coordinated water molecules [O1W and O2W, Fig. S3(*a*)]. On the other hand, Ca2 contains four phospho­nate groups (P1, P2, P3 and P4, each one from a different ligand) acting as monodentate ligands in the octahedra basal positions (by O2, O5, O7 and O11, respectively) with the O3W and O4W from two water molecules occupying the apical positions [Fig. S3(*b*)]. These pentagonal bipyramids and octahedra share two vertices (O11 and O5), generating chains in the [100] direction [Figs. 1 ▸(*b*) and S4]. Bonds between these chains are formed by the ligands, creating layers in the (001) plane in which the pyrene cores are π stacked [*d*(Py–Py) ≃ 4 Å, Fig. S5]. Each ligand is bonded to eight different Ca atoms. The P1 and P3 groups act as bidentate ligands through O1 and O2 (from P1) and through O8 and O7 (from P3), bonding to one Ca1 and one Ca2, respectively; the P2 group acts as a chelating ligand (through O5 and O6) to one Ca1, and also a bidentate ligand, bridging Ca1 and Ca2 by O5; and finally the P4 group acts as a monodentate ligand (O11), bridging one Ca1 and one Ca2 (Fig. S6). It is worth noting that during the synthesis all the eth­oxy groups were hydrolyzed, remaining as four OH groups in the coordinated ligand. This resulted in the connection of four chains, one per phospho­nate group of the ligand, where the octahedra and the pentagonal bipyramids alternate between the chains, establishing intra-layer hydrogen bonds through the coordinated water molecules (Fig. S7). Finally, the stacking of the layers generates elongated channels of *ca* 7.5 × 3.5 Å (considering van der Waals radii). These voids are occupied by free water molecules that participate in an extended hydrogen-bonding network with adjacent layers (Fig. S8). These channels account for approximately 12.8% of the unit-cell volume (according to *PLATON* calculations).

**Figure 2:**
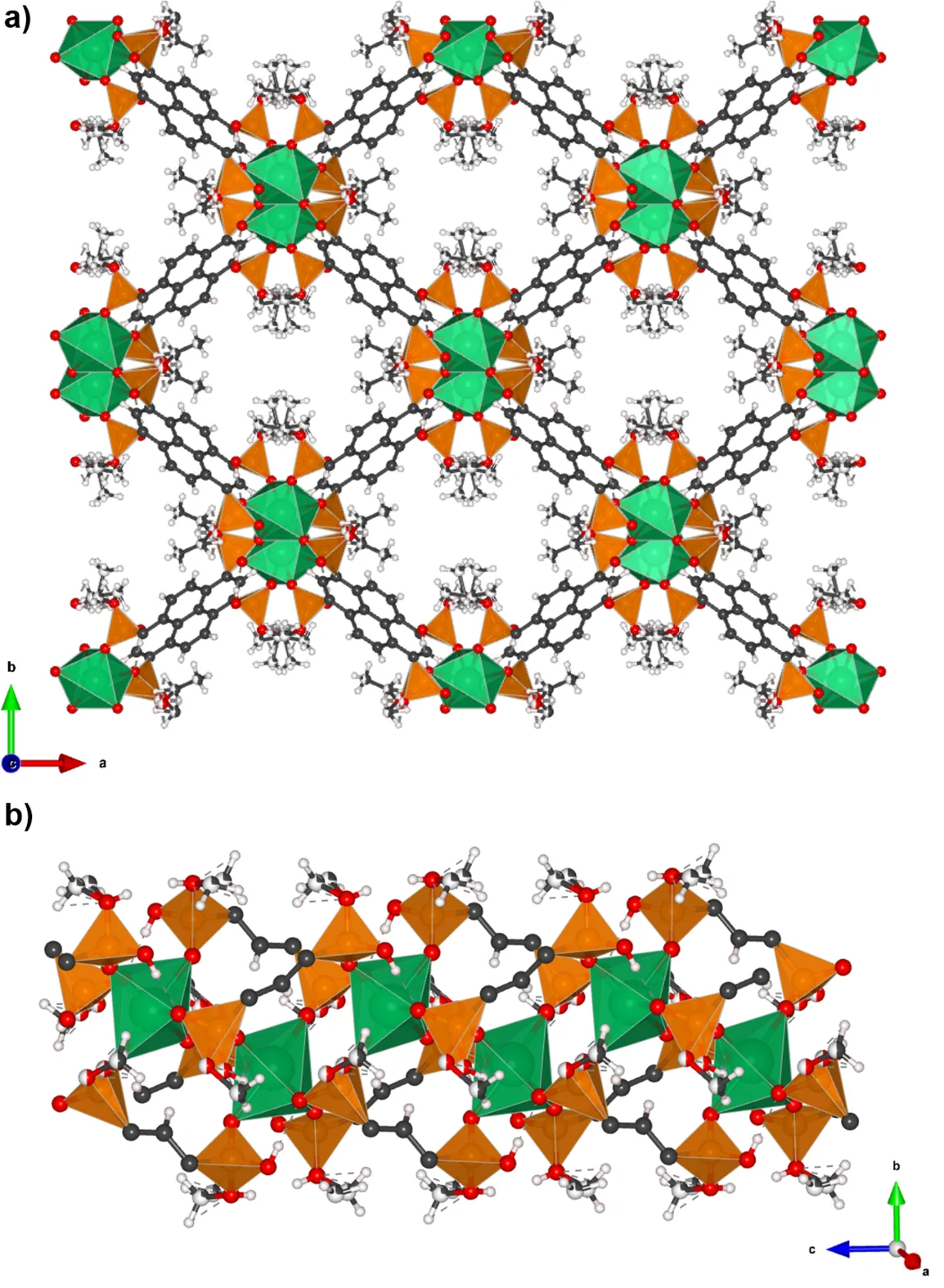
Crystal structure of IEF-34 viewed along the [001] direction (*a*) and [CaO_6_] octahedra chains in the [001] direction (*b*). Calcium, phospho­rus, oxygen, carbon and hydrogen atoms are represented in green, orange, red, black and white, respectively.

IEF-34 was obtained as small rhombohedral pale yellow crystals (100 µm in length, Fig. S9), allowing its structure to be determined using conventional SCXRD (Table 1 ▸). IEF-34, with the formula [Ca(C_20_H_20_P_4_O_12_)]*_n_* (or [Ca(Py(PO_3_H_0.5_Et_0.5_)_2_(PO_3_H_1.5_Et_0.5_)_2_)]*_n_*), crystallizes in the monoclinic space group *C*2/*c* (No. 15) with unit-cell parameters *a* = 21.6124 (1) Å, *b* = 18.4191 (8) Å, *c* = 8.3124 (4) Å, β = 108.823 (4)°, *V* = 3132.0 (3) Å^3^ and *Z* = 4. The asymmetric unit contains half a pyrene tetra­ethyl tetra­phospho­nate ligand coordinated to one Ca^2+^ cation. Unlike IEF-33, where complete hydrolysis of the phospho­nate ester groups occurs, IEF-34 retains one ethyl substituent dis­ordered between the two phospho­nate moieties of the asymmetric unit, yielding the partially hydrolyzed ligand [Py(PO_3_H_0.5_Et_0.5_)_2_(PO_3_H_1.5_Et_0.5_)_2_]^2−^. The three-dimensional structure of IEF-34 is built up from distorted [CaO_6_] octahedra [Figs. 2 ▸ and S10(*a*), and Tables 1, S6 and S7] acting as SBUs bonded by [Py(PO_3_H_0.5_Et_0.5_)_2_(PO_3_H_1.5_Et_0.5_)_2_]^2−^ with *d*(Ca1—O) distances ranging from 2.304 (4) to 2.412 (4) Å. These octahedra are interconnected by the different phos­pho­nate groups, creating a zigzag chain along the [001] direction. Each calcium center is bound to six different phospho­nate groups from neighboring ligands. Specifically, the coordination sphere is composed of four P1 and two P2 phospho­nate groups from different ligands: two O1 and two O2 from the P1 groups, and two O5 atoms from the P2 groups, where the O1 and O5 pairs of atoms are in the *cis* position and the O2 pair is in the *trans* position [Fig. S10(*a*)]. On the other hand, each ligand, where each phospho­nate group presents half a non-hydrolyzed ethyl group, is connected to six different Ca atoms: two per P1 group (O1 and O2) and one per P2 group (O5), as illustrated in Fig. S10(*b*). A notable feature of IEF-34 is the presence of one-dimensional channels running parallel to the [001] direction. Two types of apertures can be identified: narrower channels (7.5 × 2 Å) in between disordered ethyl groups and larger cavities of *ca* 10 × 10 Å delimited by the pyrene cores along the [001] direction with the ethyl groups located inside (considering the van der Waals radii). These channels represent 13.2% of the unit-cell volume according to solvent-accessible volume calculations in *PLATON*, being the permanent porosity further confirmed by N_2_ and CO_2_ gas sorption experiments (see below).

### 3.2. Physicochemical characterization

The purity of the polycrystalline samples of both IEF-33 and IEF-34 was confirmed by powder X-ray diffraction (PXRD) combined with chemical analysis. (IEF-33: theoretical (%) Ca 11.06, P 17.10, C 26.52, H 3.34, O 41.96; experimental (%) Ca 11.2 ± 0.6, P 18.8 ± 0.9, C 27.26 ± 0.08, H 2.96 ± 0.08. IEF-34: theoretical (%) Ca 6.50, P 20.10, C 38.97, H 3.27, O 31.15; experimental (%) Ca 6.8 ± 0.3, P 19.0 ± 0.9, C 39.4 ± 0.1, H 3.66 ± 0.01; see the supporting information for further details). Comparison of the position and intensity of the main reflections in the experimental PXRD patterns with the simulated ones confirmed the successful formation of the phases (Fig. S11), while a Pawley profile fitting (Fig. S12 and Table S7) showed the absence of other crystalline impurities. The ligand connectivity was confirmed by Fourier-transform infrared spectroscopy (FTIR, Fig. S13), where the elimination of the νC_aliphatic_—H, δC_aliphatic_—H and νP—O—C bands (at 2900–2982, 1370–1478 and 798 cm^−1^, respectively) (Pretsch *et al.*, 2009 ▸) was observed in IEF-33 due to the hydrolysis of the eth­oxy groups of the phospho­nate (while those signals are still present in IEF-34), combined with a shift in the main νP—O and νP=O bands with respect to the Py(PO_3_Et_2_)_4_ ligand (from 1247 and 1217 cm^−1^ to 1127 cm^−1^ for IEF-33 and to 1216 and 1175 cm^−1^ for IEF-34; and from 1012 and 957 to 1017 and 913 for IEF-33 and to 1027 and 944 cm^−1^ for IEF-34).

**Figure 3:**
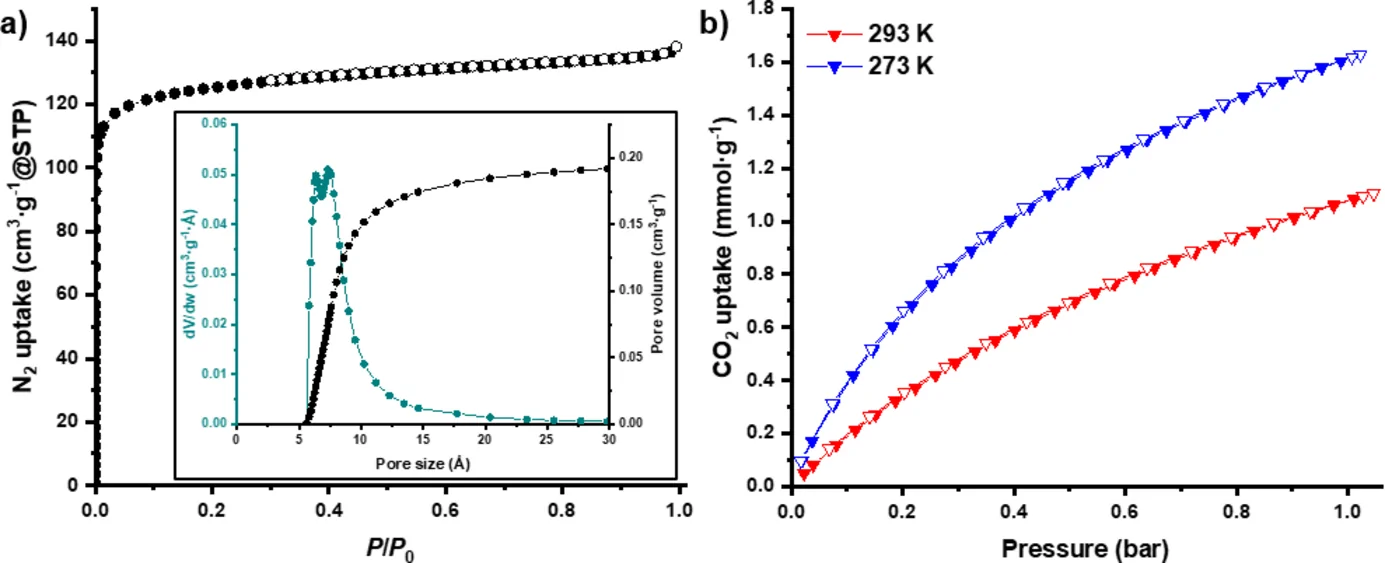
(*a*) N_2_ sorption isotherm at 77 K and Horvath–Kawazoe pore size distribution (inset), and (*b*) CO_2_ sorption isotherms at 273 K (blue) and 293 K (red) of IEF-34. Adsorption and desorption points are represented by solid and open symbols, respectively.

The potential permanent porosity of both IEF-33 and IEF-34 was evaluated by N_2_ and CO_2_ sorption isotherms. IEF-33 showed almost no N_2_ or CO_2_ uptake (Fig. S14) despite the *a priori* accessible porosity according to the crystallographic data (pore dimensions: 7.5 × 3.5 Å versus the kinetic diameters of N_2_ and CO_2_ of 3.6 and 3.3 Å, respectively). This behavior can be rationalized by considering the phase transition occurring upon activation (see *Thermal and chemical stability*[Sec sec3.3] below). On the other hand, IEF-34 presented permanent porosity accessible to N_2_ and CO_2_ (Fig. 3 ▸), with an *S*_BET_ surface area of 491.3 ± 0.5 m^2^ g^−1^ and a total pore volume (*V*_p_) of 0.19 cm^3^ g^−1^ according to the N_2_ isotherm [Fig. 3 ▸(*a*)]. The Horvath–Kawazoe pore size distribution [inset to Fig. 3 ▸(*a*)] showed a broad pore size distribution between 6–7.5 Å, in good agreement with the crystallographic data considering the free rotation inside the pore and the disordered nature of the eth­oxy groups and their partial occupancy. Finally, IEF-34 showed a CO_2_ uptake of 1.6 and 1.1 mmol g^−1^ at 1 bar [at 273 and 293 K, respectively; Fig. 3 ▸(*b*)], in line with previously reported P-MOFs (Salcedo-Abraira *et al.*, 2021 ▸; Taddei *et al.*, 2014 ▸; Serrano-Nieto *et al.*, 2026 ▸; Glavinović *et al.*, 2022 ▸) but still far from the benchmarked Mg carboxyl­ate MOF-74, which has the record for CO_2_ uptake in the field of MOFs (8 mmol g^−1^ at 296 K and 1 bar; Li *et al.*, 2020 ▸).

### 3.3. Thermal and chemical stability

In order to assess the thermal stability of the two MOFs, thermogravimetric analyses coupled with differential scanning calorimetry (TGA–DSC) and variable-temperature PXRD (VTPXRD) experiments were carried out. TGA of IEF-33 showed three consecutive weight losses associated with the removal of water molecules (Fig. S15). The first weight loss (from RT to 80 °C) of 7.7% corresponds to the departure of three free water molecules located between the layers (theoretical loss 7.45%). A second weight loss of 6.1% from 80 to 100 °C is attributed to the removal of three of the four coordinated water molecules (theoretical loss 7.45%). The remaining coordinated water molecule seems to be released more gradually from 100 to 200 °C, resulting in an additional weight loss of 2.5% (theoretical loss 2.48%). After complete dehydration, a plateau is observed up to 400 °C where IEF-33 is oxidized prior to decomposition (Fig. S15). This oxidation may be attributed to the formation of phosphates from the phospho­nate groups (Howell, 2022 ▸). VTPXRD (Fig. S16) revealed an initial phase transition as a consequence of water departure starting at 30 °C and finishing at 150 °C, in line with the TGA. This transition was found to be irreversible, as the original structure was not recovered after cooling back to room temperature or following subsequent exposure to water, indicating a permanent structural rearrangement upon dehydration (Fig. S17). This phase remained stable up to 360 °C, where it evolved due to oxidation prior to the decomposition. Note that it was not possible to identify the nature of the final residue because of its amorphous nature, hindering complete compositional characterization. These observations are consistent with the absence of N_2_ and CO_2_ uptake from IEF-33 despite its *a priori* structural porosity.

On the other hand, and consistent with its rigid 3D network and permanent porosity, IEF-34 showed a progressive initial weight loss (∼5%) from room temperature to *ca* 120 °C that might correspond to some residual solvent molecules trapped within the pores during the synthesis. Then a plateau is observed up to 300 °C, where IEF-34 started to decompose (Fig. S18; note that the final residue was not identified). VTPXRD showed no significant structural evolution up to 270 °C, where the structure collapsed due to decomposition (Fig. S19), in agreement with the TGA data.

The chemical stability of IEF-33 and IEF-34 was evaluated by suspending the materials (5 mg ml^−1^) under stirring for 24 h in a series of organic solvents (methanol, MeOH; ethanol, EtOH; aceto­nitrile, ACN; tetra­hydro­furan, THF; *N*,*N*′-di­methyl­formamide, DMF; di­methyl­sulfoxide, DMSO; CH_2_Cl_2_; hexane; octane; and toluene). In addition, their hydrolytic stability was evaluated over a broad range of pH. After treatment, the solids were recovered and their structural integrity was evaluated by PXRD. IEF-33 retained its crystallinity over the 2 > pH > 10 range, losing its crystallinity at pH 13 (Fig. S20). Upon exposure to different organic solvents, IEF-33 preserved its crystalline structure with only minor changes in peak positions and relative intensities in most cases. The only exception was for DMSO, where the appearance of additional diffraction peaks suggested the formation of a secondary phase. This behavior is probably associated with the replacement of the interlayer water molecules by DMSO, leading to a partial structural reorganization of the layered framework (Fig. S21).

On the other hand, IEF-34 exhibited remarkable hydrolytic structural stability, maintaining its crystallinity across almost the entire pH range (pH 1–12). Under strongly basic conditions (pH 14), the material was transformed into Ca(OH)_2_ (JCPDS Card No. 87-s0315, Fig. S22). IEF-34 presented negligible changes in PXRD patterns when exposed to the various organic solvents (Fig. S23), showing superior stability in organic media to IEF-33. We hypothesize that the enhanced chemical stability of IEF-34 relative to IEF-33 originates from the different coordination environment of the Ca^2+^ centers; specifically, while IEF-33 features labile coordinated water molecules, the calcium atoms in IEF-34 are exclusively coordinated by phospho­nate groups, resulting in a more robust inorganic framework that effectively resists chemical degradation.

Complementary gravimetric measurements after solvent treatment revealed solvent-dependent mass variations (Fig. S24). The largest mass loss was observed after exposure to deionized water (>40 wt%), whereas hydro­carbon solvents led to more moderate values (<30 wt%). Intermediate mass changes were measured for THF and toluene (<20 wt%), while ethanol, aceto­nitrile, DMF and DMSO exhibited the lowest variations (<10 wt%). Interestingly, despite these differences in mass variation, PXRD patterns confirmed that the crystalline framework was preserved after treatment in all cases (except for the partial structural reorganization observed in DMSO).

### 3.4. Optoelectronic properties

**Figure 4:**
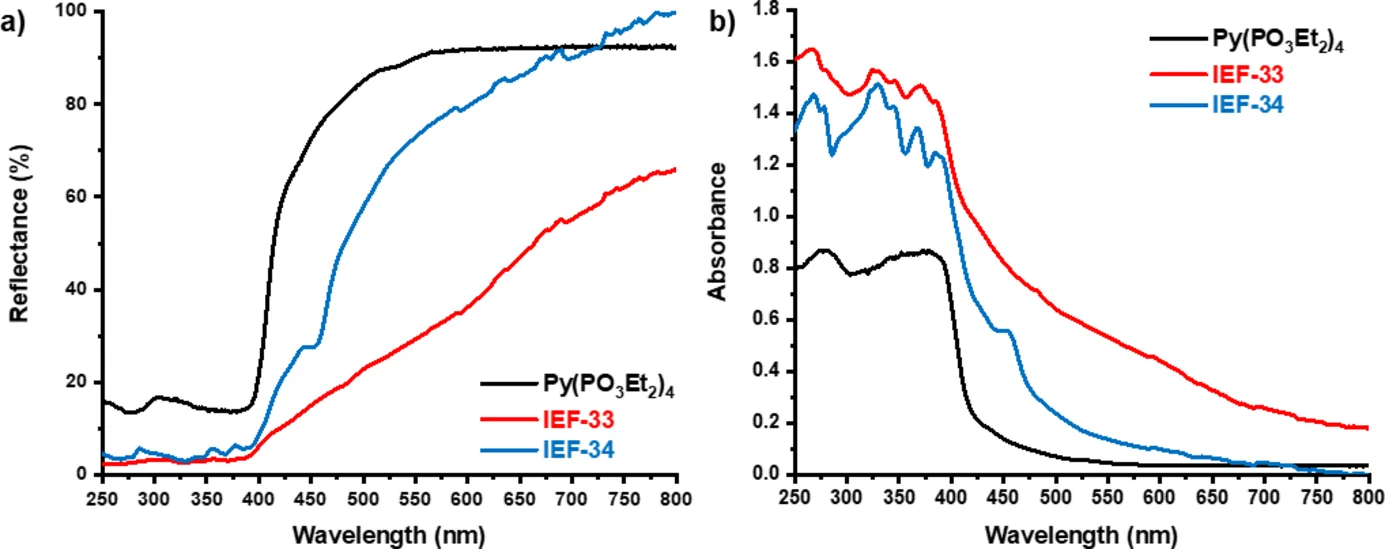
Solid-state UV–Vis diffuse reflectance (*a*) and absorption (*b*) spectra of the Py(PO_3_Et_2_)_4_ ligand (black), IEF-33 (red) and IEF-34 (blue).

Given the promising photoactive properties previously reported for pyrene-tetra­phospho­nate-based materials (Serrano-Nieto *et al.*, 2025 ▸; Salcedo-Abraira, Serrano-Nieto *et al.*, 2023 ▸), the optoelectronic properties of IEF-33 and IEF-34 were investigated by solid-state diffuse reflectance UV–Vis spectroscopy. Identifying their light-absorption behavior is particularly relevant for assessing their potential in applications such as photocatalysis, optoelectronics and solar energy conversion. These MOFs showed light absorption across almost all the visible range (Fig. 4 ▸), with a broad absorption maximum ranging from 250 to 400 nm due to the aromatic pyrene core, and in line with the optoelectronic properties of the ligand. Between 400 and 800 nm, IEF-33 presented stronger absorption than IEF-34, absorbing within the whole range (while IEF-34 started to absorb at *ca* 650 nm). These differences in terms of optical properties are probably related to the pyrene core π–π stacking present in the IEF-33 structure, which is also responsible for the different colors of the two materials (brown for IEF-33 and pale yellow for IEF-34), and are also reflected in the estimated optical band gap: 2.51 versus 2.62 eV for IEF-33 and IEF-34, respectively (Fig. S25), which both correspond to indirect transitions.

The combination of permanent porosity (in IEF-34), broad visible light absorption and high chemical stability across a wide pH range suggests that these calcium-based frameworks could be of interest for several applications. For instance, while the CO_2_ uptake at low pressures remains modest and further selectivity studies are required, the robust 3D structure and high stability of IEF-34 provide a baseline for exploring gas sorption in chemically demanding environments (Xian *et al.*, 2021 ▸). Furthermore, the photoactive pyrene core, coupled with the observed narrow band gaps, indicates that these materials may serve as platforms for the (photo)catalytic degradation of organic pollutants or solar-to-chemical energy conversion (Serrano-Nieto *et al.*, 2025 ▸; Lustig & Li, 2018 ▸). Finally, the inherent biocompatibility of the calcium-phospho­nate backbone opens up potential avenues for future exploration in biomedical fields, such as drug delivery or biosensing (Alvarez *et al.*, 2013 ▸; Liu *et al.*, 2012 ▸).

## 4. Conclusions

Two novel calcium-based P-MOFs were solvothermally synthesized using a photoactive pyrene tetra­phospho­nate ligand. Single-crystal X-ray diffraction revealed that differences in ligand hydrolysis led to markedly different architectures: hydrogen-bonded 2D coordination layers in IEF-33 and a 3D porous network with 1D channels in IEF-34. These MOFs have open structures with free water molecules (IEF-33) or voids (IEF-34), showing their potential porosity. Furthermore, IEF-34 had a CO_2_ adsorption of *ca* 1.6 mmol g^−1^ at 273 K, which is in the range of the highest values reported for P-MOFs. Additionally, both materials showed moderate thermal stability (up to 350 and 270 °C for IEF-33 and IEF-34, respectively) and good chemical stability in aqueous and organic solvents, which when combined with their optoelectronic properties (band gaps of 2.51 and 2.62 eV) make IEF-33 and IEF-34 potential candidates for several applications [*e.g.* molecule adsorption, sensing, (photo)catalysis]. Specifically, the permanent porosity of IEF-34 opens up potential avenues for energy-efficient gas separation in harsh environments and future biomedical exploration. More broadly, this work expands the still-limited family of porous calcium P-MOFs and provides new insights into the crystal engineering of sustainable MOFs.

## 5. Related literature

The following reference is cited in the supporting information: Bernhardt *et al.* (2006 ▸).

## Supplementary Material

Crystal structure: contains datablock(s) PyPO3Et2-4, IEF-33, IEF-34. DOI: 10.1107/S2052252526009875/hay5002sup1.cif

Structure factors: contains datablock(s) PyPO3Et2-4. DOI: 10.1107/S2052252526009875/hay5002PyPO3Et2-4sup2.hkl

Structure factors: contains datablock(s) IEF-33. DOI: 10.1107/S2052252526009875/hay5002IEF-33sup3.hkl

Structure factors: contains datablock(s) IEF-34. DOI: 10.1107/S2052252526009875/hay5002IEF-34sup4.hkl

Supporting results and additional experimental details. DOI: 10.1107/S2052252526009875/hay5002sup5.pdf

CCDC references: 2548440, 2548343, 2248686

## Figures and Tables

**Table 1:** Crystal data and structure refinement for Py(PO_3_Et_2_)_4_, IEF-33 and IEF-34

	Py(PO_3_Et_2_)_4_	IEF-33	IEF-34
CCDC number	2548440	2548343	2248686
Empirical formula	C_32_H_46_O_12_P_4_	Ca_2_C_16_H_24_O_19_P_4_	CaC_20_H_20_O_12_P_4_
Formula weight	746.57	724.39	616.32
Temperature (K)	296.15	100.00	295.7 (4)
Crystal system	triclinic	triclinic	monoclinic
Space group	*P* 1	*P* 1	*C*2/*c*
*a* (Å)	10.0133 (4)	8.4993 (3)	21.6124 (1)
*b* (Å)	10.1039 (4)	12.5656 (9)	18.4191 (8)
*c* (Å)	10.9607 (4)	12.5832 (1)	8.3124 (4)
α (°)	66.7250 (1)	103.252 (7)	90
β (°)	74.4070 (1)	98.700 (5)	108.823 (4)
γ (°)	89.869 (2)	97.446 (4)	90
Volume (Å^3^)	974.40 (7)	1274.05 (2)	3132.0 (3)
*Z*	1	2	4
ρ_calc_ (g cm^−3^)	1.272	1.888	1.307
μ (mm^−1^)	0.249	1.212	0.455
*F*(000)	394.0	744.0	1264.0
Crystal size (mm)	0.1 × 0.003 × 0.003	0.1 × 0.01 × 0.01	0.1 × 0.05 × 0.02
Radiation	Mo *K*α (λ = 0.71073)	Synchrotron (λ = 0.82656)	Mo *K*α (λ = 0.71073)
2θ range for data collection (°)	4.23 to 56.664	3.93 to 52.762	6.372 to 50.052
Index ranges	−13 ≤ *h* ≤ 13, −13 ≤ *k* ≤ 13, −14 ≤ *l* ≤ 14	−9 ≤ *h* ≤ 9, −12 ≤ *k* ≤ 12, 0 ≤ *l* ≤ 13	−25 ≤ *h* ≤ 25, −21 ≤ *k* ≤ 21, −9 ≤ *l* ≤ 9
Reflections collected	49829	10741	36413
Independent reflections	4852 (*R*_int_ = 0.0729, *R*_sigma_ = 0.0392)	3082 (*R*_int_ = 0.2687, *R*_sigma_ = 0.2076)	2761 (*R*_int_ = 0.0882, *R*_sigma_ = 0.0434)
Data/restraints/parameters	4852/179/241	3082/1/377	2761/179/216
Goodness-of-fit on *F*^2^	1.042	1.040	1.102
Final *R* factors [*I* ≥ 2σ(*I*)]	*R*_1_ = 0.0711, *wR*_2_ = 0.2121	*R*_1_ = 0.1205, *wR*_2_ = 0.3061	*R*_1_ = 0.0615, *wR*_2_ = 0.1560
Final *R* factors (all data)	*R*_1_ = 0.0907, *wR*_2_ = 0.2414	*R*_1_ = 0.1757, *wR*_2_ = 0.3234	*R*_1_ = 0.0945, *wR*_2_ = 0.1726
Largest difference peak/hole (e Å^−3^)	0.46/−0.35	1.67/−0.70	0.40/−0.29

## Data Availability

The data supporting this article are included as part of the supporting information. Crystal data (excluding structure factors) have also been deposited with the Cambridge Crystallographic Data Centre as supplementary publications No. 2548440, 2548343 and 2248686 for Py(PO_3_Et_2_)_4_, IEF-33 and IEF-34, respectively. These data are provided free of charge by the joint Cambridge Crystallographic Data Centre and Fachinformationszentrum Karlsruhe Access Structures service.
