# Supplementary material for: Two calcium pyrene phospho­nate frameworks with contrasting dimensionality and porosity
Source: IUCrJ. 2026 Nov 6;13(Pt 6):843–51. doi: 10.1107/S2052252526009875 (PMC13763894; doi:10.1107/S2052252526009875)
Supplement: Supplementary file 1 [file m-13-00843-sup5.pdf]

IUCrJ

Volume 13 (2026)

Supporting information for article:

Two calcium pyrene phosphonate frameworks with contrasting dimensionality and porosity

Pablo Salcedo-Abraira, Rubén Serrano-Nieto, Sergio Carrasco, Catalina Biglione, Inhar Imaz, Thomas Devic, Daniel Maspoch and Patricia Horcajada

S1. Synthesis of the $Py(PO_3Et_2)_4$ linker

The lab-made $Py(PO_3Et_2)$ linker was synthesized following the Scheme S1:

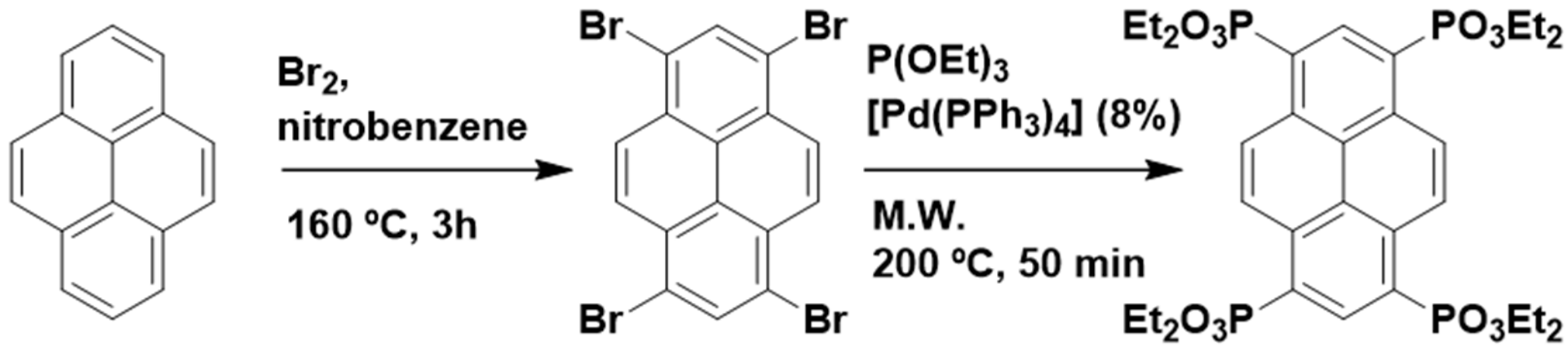

Scheme S1. Synthetic route towards the preparation of $Py(PO_3Et_2)_4$.

Py(Br)4:

$Py(Br)_4$ was synthesized according to a published work with slight modifications.[1] In a 250 mL round-bottom flask, 2 g (9.88 mmol) of pyrene were dissolved in 60 mL of nitrobenzene at room temperature (RT) under constant magnetic stirring. 6.48 g of Br_2 (40.56 mmol) were added, and the resulting mixture was heated with a condenser at 160 ºC for 3 h. After cooling to ambient temperature, acetone was added (approximately 50 mL) and the resulting yellowish $Py(Br)_4$ solid was filtered, washed with acetone and dried at ambient conditions. Yield = 97%.

Py(PO3Et2)4:

In a 30 mL microwave reactor, 0.6 g of $Py(Br)_4$ (1.16 mmol), 0.1063 g of $[Pd(PPh_3)_4]$ (8% based on the mol of $Py(Br)_4$, 0.092 mmol) and 6 mL of triethyl phosphite were added and stirred for 1 min. Then, the mixture was put inside the microwave and heated following the temperature program: i) heating up to 200 ºC for 5 min; ii) keeping at 200 ºC for 50 min; and iii) cooling down to 60 ºC.

After the reaction, the reactor was placed overnight at ambient conditions, leading to the crystallization of the desired compound. The reactor was filled with petroleum ether, sonicated for 1 min and the resulting yellow solid was filtered. The obtained crystals, denoted $Py(PO_3Et_2)_4$, was washed again by suspending it in 30 mL of petroleum ether and sonicated for a few minutes in order to remove some residual triethyl phosphite and impurities. Finally, it was filtered and dried under ambient conditions. Yield = 92%. Before the sonication step, suitable single-crystals were collected and measured by SCXRD (Table1, Tables S1-2 and Figure S1).

S2. Chemical analysis.

Chemical analyses were conducted by light elemental analysis and ICP-OES. *Prior* to the IEF-34 analyses, the material was dried in an oven at 100 ºC in order to remove the potential guest molecules inside the pores.

IEF-33 – $\{[Ca_2(C_{16}H_{10}P_4O_{12})(H_2O)_4]\cdot 3H_2O\}_n$

Theo. (%): Ca 11.06; P 17.10; C 26.52; H 3.34; O 41.96

Exp. (%): Ca 11.2 ± 0.6; P 18.8 ± 0.9; C 27.26 ± 0.08; H 2.96 ± 0.08

IEF-34 – $[Ca(C_{20}H_{20}P_4O_{12})]_n$

Theo. (%): Ca 6.50; P 20.10; C 38.97; H 3.27; O 31.15

Exp. (%): Ca 6.8 ± 0.3; P 19.0 ± 0.9; C 39.4 ± 0.1; H 3.66 ± 0.01

S3. Chemical stability experiments.

The chemical stability of the materials was assessed by suspending 10 mg of solid in 2 mL of solvent under stirring at room temperature during 24 h. Then, the solids were recovered by centrifugation and washed twice with ethanol and dried at 80 ºC for 24 h. The obtained solid was weighted and further analyzed by PXRD.

Table S1 Bond lengths of $Py(PO_3Et_2)_4$.

Atom	Atom	Length/Å	Atom	Atom	Length/Å
P1	O3	1.571(2)	C4	C7	1.430(3)
P1	O2	1.559(2)	C4	C3	1.402(3)
P1	O1	1.461(2)	C7	C8	1.350(4)
P1	C3	1.808(2)	C3	C2	1.387(3)
P2	O5	1.564(3)	C6	C8[1]	1.432(3)
P2	O4	1.461(3)	C6	C1	1.407(3)
P2	O6	1.567(3)	O6	C15A	1.319(2)
P2	C1	1.797(3)	O6	C15B	1.391(2)
O3	C11	1.451(4)	C1	C2	1.398(3)
O2	C9	1.442(4)	C9	C10	1.382(7)
O5	C13	1.423(6)	C11	C12	1.347(7)
C5	C5[1]	1.439(4)	C13	C14	1.380(8)
C5	C4	1.426(3)	C15A	C16A	1.415(1)
C5	C6	1.424(3)	C15B	C16B	1.390(2)

[1]1-X,2-Y,1-Z

Table S2 Bond angles of Py(PO_3Et_2)$_4$.

Atom	Atom	Atom	Angle/°	Atom	Atom	Atom	Angle/°
O3	P1	C3	106.19(1)	C3	C4	C7	122.6(2)
O2	P1	O3	102.14(1)	C8	C7	C4	121.9(2)
O2	P1	C3	101.95(1)	C4	C3	P1	121.67(2)
O1	P1	O3	114.16(1)	C2	C3	P1	118.03(2)
O1	P1	O2	116.28(2)	C2	C3	C4	120.3(2)
O1	P1	C3	114.55(1)	C5	C6	C8[1]	118.3(2)
O5	P2	O6	99.53(2)	C1	C6	C5	119.0(2)
O5	P2	C1	107.14(1)	C1	C6	C8[1]	122.8(2)
O4	P2	O5	115.42(2)	C15A	O6	P2	119.4(7)
O4	P2	O6	116.45(2)	C15B	O6	P2	129.2(8)
O4	P2	C1	111.17(1)	C7	C8	C6[1]	121.7(2)
O6	P2	C1	106.01(2)	C6	C1	P2	124.26(2)
C11	O3	P1	120.9(2)	C2	C1	P2	115.81(2)
C9	O2	P1	120.1(2)	C2	C1	C6	119.9(2)
C13	O5	P2	118.3(3)	C3	C2	C1	121.5(2)
C4	C5	C5[1]	119.8(2)	C10	C9	O2	109.5(4)
C6	C5	C5[1]	120.0(2)	C12	C11	O3	112.5(4)
C6	C5	C4	120.2(2)	C14	C13	O5	116.6(5)
C5	C4	C7	118.3(2)	O6	C15A	C16A	117.1(1)
C3	C4	C5	119.1(2)	C16B	C15B	O6	123(2)

[1]1-X,2-Y,1-Z

Table S3 Selected bond lengths of IEF-33.

Atom	Atom	Length/Å	Atom	Atom	Length/Å
Ca1	O1	2.390(1)	Ca2	O4W	2.325(2)
Ca1	O11[3]	2.543(9)	Ca2	O3W	2.415(1)
Ca1	O5[4]	2.569(1)	Ca2	O11[2]	2.336(7)
Ca1	O8[5]	2.392(1)	Ca2	O5[3]	2.364(7)
Ca1	O6[4]	2.761(1)	Ca2	O2[1]	2.301(9)
Ca1	O2W	2.362(1)	Ca2	O7	2.279(1)
Ca1	O1W	2.449(1)			

[1]+X,-1+Y,+Z; [2]-X,-Y,1-Z; [3]1-X,1-Y,1-Z; [4]1-X,2-Y,1-Z; [5]1+X,1+Y,+Z

Table S4 Selected bond angles of IEF-33.

Atom	Atom	Atom	Angle/°	Atom	Atom	Atom	Angle/°
O1	Ca1	$O11^{3}$	85.4(3)	O4W	Ca2	O3W	173.8(3)
O1	Ca1	$O12^{3}$	70.9(3)	O4W	Ca2	$O11^{2}$	96.4(4)
O1	Ca1	$O5^{5}$	76.0(3)	O4W	Ca2	$O5^{3}$	93.9(4)
O1	Ca1	$O8^{4}$	100.5(4)	$O11^{2}$	Ca2	O3W	87.7(4)
O1	Ca1	$O6^{5}$	130.2(3)	$O11^{2}$	Ca2	$O5^{3}$	169.5(5)
O1	Ca1	O1W	91.7(3)	$O5^{3}$	Ca2	O3W	81.9(4)
$O11^{3}$	Ca1	$O12^{3}$	53.1(3)	$O2^{1}$	Ca2	O4W	92.2(4)
$O11^{3}$	Ca1	$O5^{5}$	153.8(4)	$O2^{1}$	Ca2	O3W	83.5(4)
$O11^{3}$	Ca1	$O6^{5}$	136.9(2)	$O2^{1}$	Ca2	$O11^{2}$	84.6(3)
$O5^{5}$	Ca1	$O12^{3}$	133.0(3)	$O2^{1}$	Ca2	$O5^{3}$	92.9(3)
$O5^{5}$	Ca1	$O6^{5}$	54.8(3)	O7	Ca2	O4W	93.3(5)
$O8^{4}$	Ca1	$O11^{3}$	76.3(3)	O7	Ca2	O3W	90.8(4)
$O8^{4}$	Ca1	$O12^{3}$	128.7(3)	O7	Ca2	$O11^{2}$	98.0(3)
$O8^{4}$	Ca1	$O5^{5}$	89.0(3)	O7	Ca2	$O5^{3}$	83.4(3)
$O8^{4}$	Ca1	$O6^{5}$	74.1(3)	O7	Ca2	$O2^{1}$	173.6(5)
$O8^{4}$	Ca1	O1W	160.8(4)				
$O6^{5}$	Ca1	$O12^{3}$	149.6(4)				
O2W	Ca1	O1	154.2(4)				
O2W	Ca1	$O11^{3}$	80.7(3)				
O2W	Ca1	$O12^{3}$	83.4(3)				
O2W	Ca1	$O5^{5}$	123.2(4)				
O2W	Ca1	$O8^{4}$	97.2(4)				
O2W	Ca1	$O6^{5}$	72.9(3)				
O2W	Ca1	O1W	76.8(4)				

Atom	Atom	Atom	Angle/°	Atom	Atom	Atom	Angle/°
O1W	Ca1	O11[3]	119.9(3)				
O1W	Ca1	O12[3]	69.3(3)				
O1W	Ca1	O5[5]	79.6(3)				
O1W	Ca1	O6[5]	86.7(3)				

[1]+X,-1+Y,+Z; [2]-X,-Y,1-Z; [3]1-X,1-Y,1-Z; [4]1+X,1+Y,+Z; [5]1-X,2-Y,1-Z

Table S5 Selected bond lengths of IEF-34.

Atom	Atom	Length/Å	Atom	Atom	Length/Å
Ca1	O1	2.304(4)	Ca1	O2[2]	2.412(4)
Ca1	O5[1]	2.345(4)			
P1	O2	1.496(4)	P2	O5	1.463(4)
P1	O1	1.466(4)	P2	O4	1.562(5)
P1	O3	1.577(5)	P2	O6	1.521(6)

[1]-1-X,1-Y,-Z; [2]+X,1-Y,1/2+Z

Table S6 Selected bond angles of IEF-34.

Atom	Atom	Atom	Angle/°	Atom	Atom	Atom	Angle/°
O5[1]	Ca1	O5[2]	82.2(2)	O2[4]	Ca1	O2[3]	155.5(2)
O5[2]	Ca1	O2[3]	82.33(1)	O1[5]	Ca1	O5[2]	160.92(1)
O5[2]	Ca1	O2[4]	79.28(1)	O1	Ca1	O5[2]	92.18(2)
O1	Ca1	O2[3]	81.90(1)	O1	Ca1	O1[5]	98.6(2)
O1	Ca1	O2[4]	114.68(1)				

[1]-1-X,1-Y,-Z; [2]+X,1-Y,-1/2+Z; [3]+X,1-Y,1/2+Z; [4]-1-X,1-Y,-1-Z

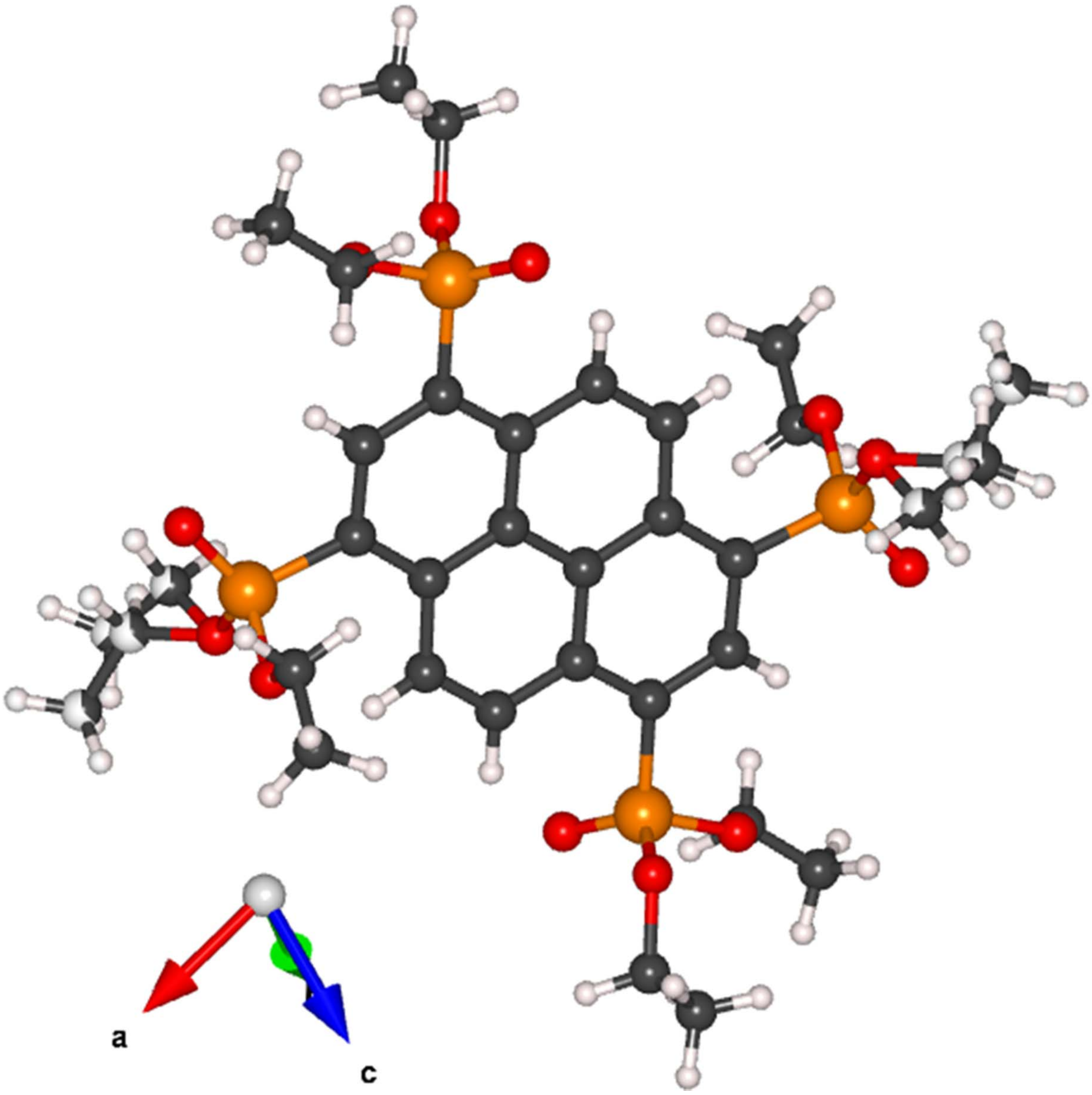

Figure S1 Crystal structure of the $Py(PO_3Et_2)_4$ ligand. Phosphorus, oxygen, carbon and hydrogen atoms are represented in orange, red, black and white; respectively. The disorder of the ethoxy groups is represented by partial colouring the atoms as a function of the % of occupation.

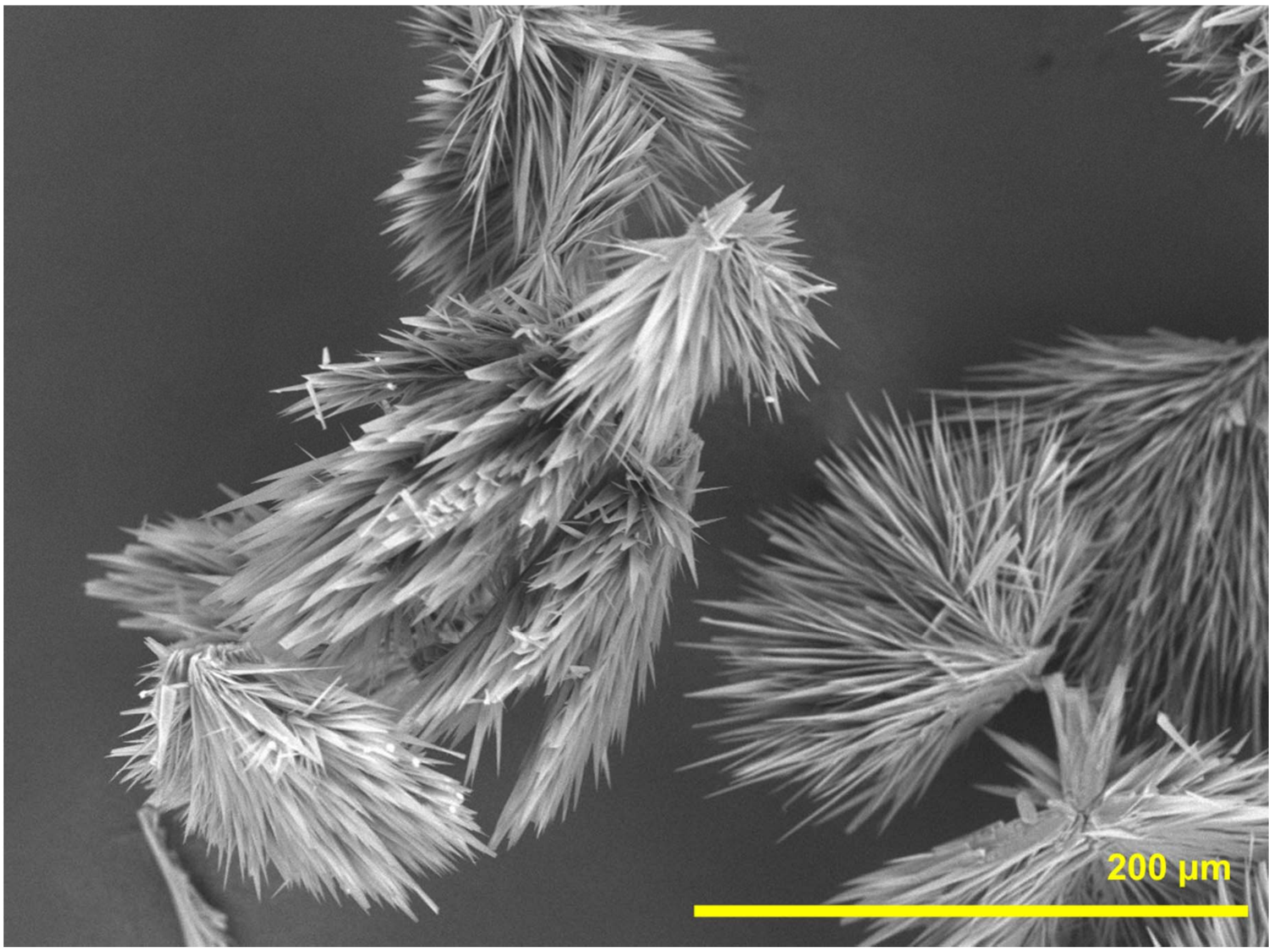

Figure S2 SEM image of IEF-33.

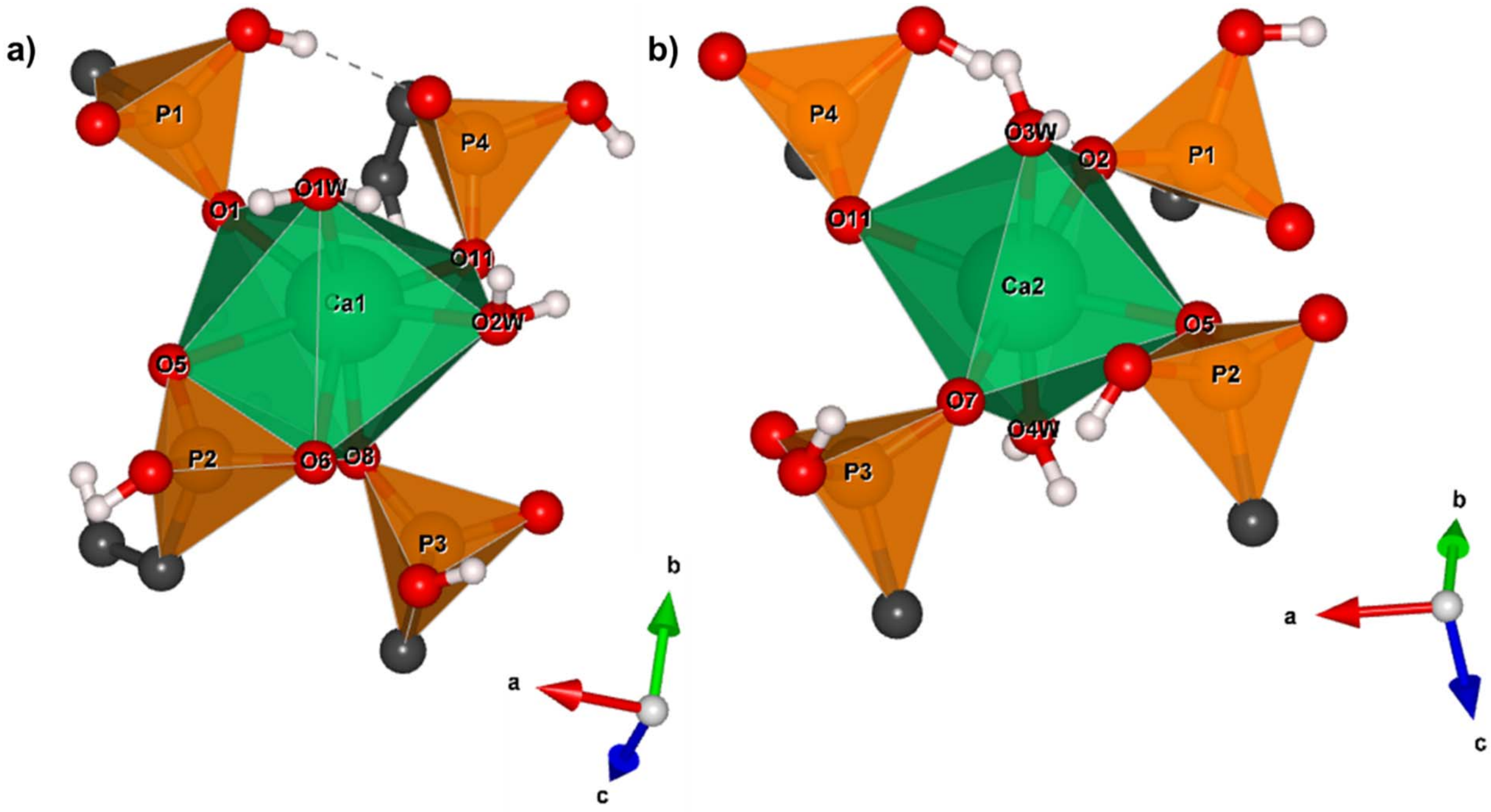

Figure S3 IEF-33 coordination spheres for Ca1 (a) and Ca2 (b). Calcium, phosphorus, oxygen, carbon and hydrogen atoms are represented in green, orange, red, black and white; respectively.

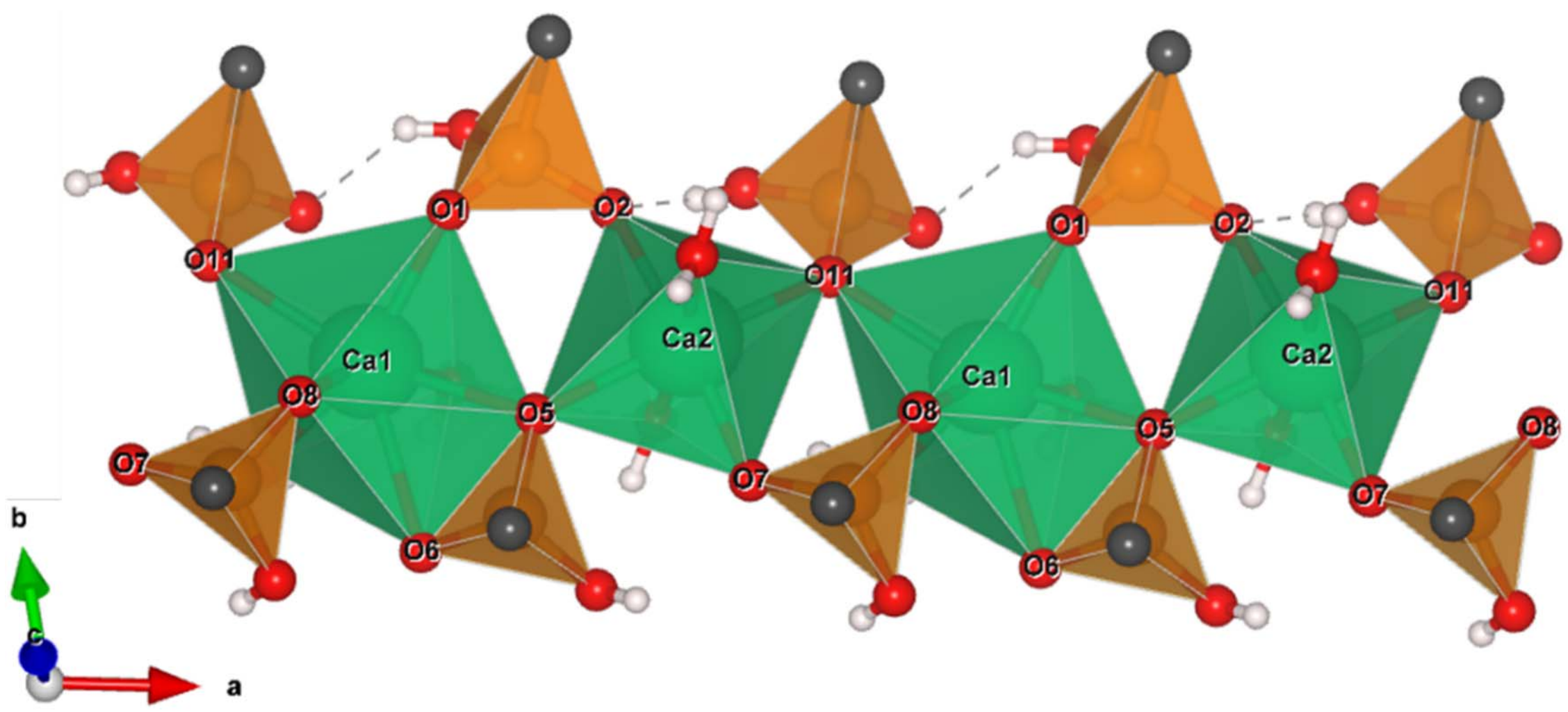

Figure S4 IEF-33 SBU connectivity. Calcium, phosphorus, oxygen, carbon and hydrogen atoms are represented in green, orange, red, black and white; respectively.

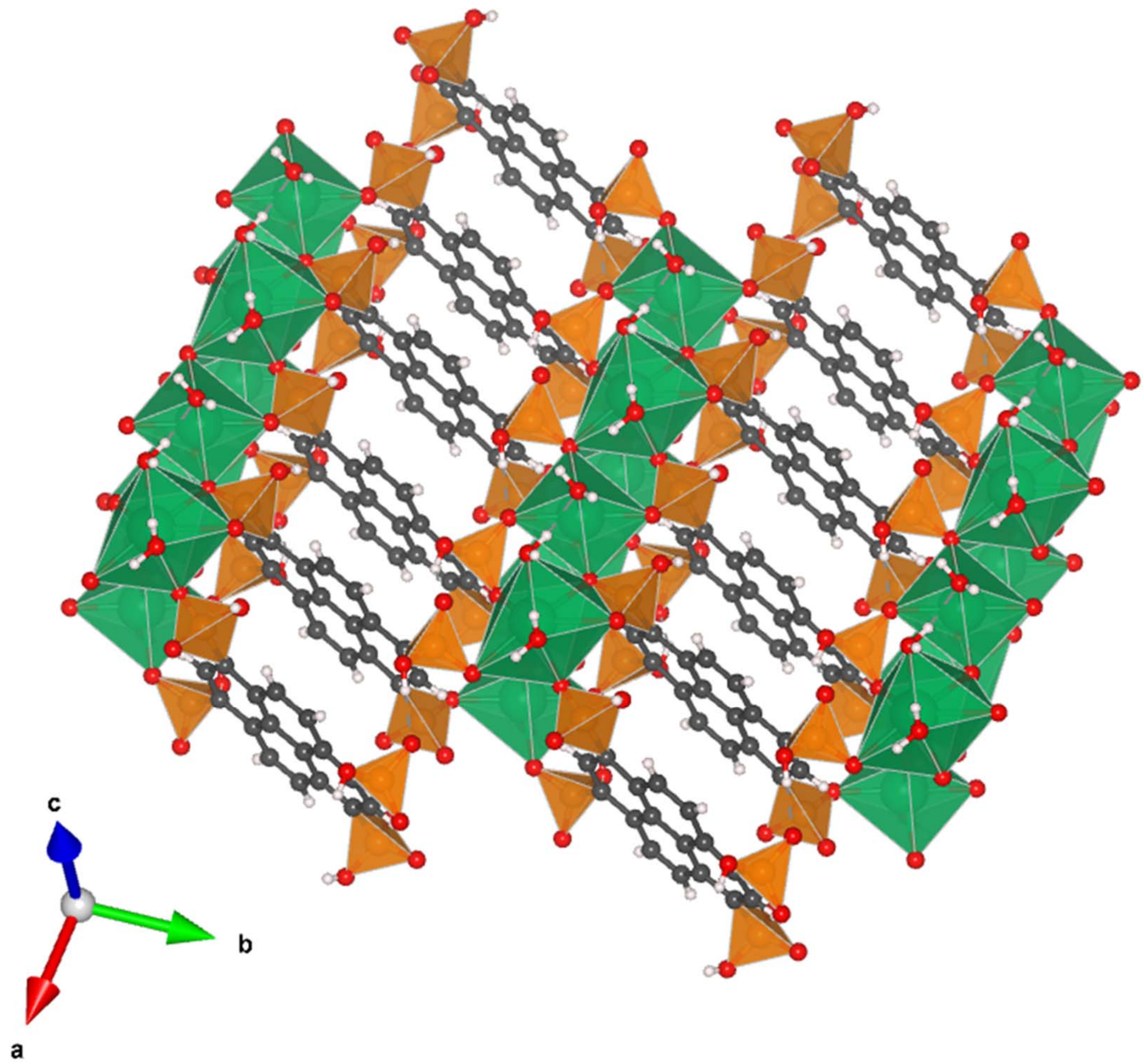

Figure S5 View of the IEF-33 layer. Calcium, phosphorus, oxygen, carbon and hydrogen atoms are represented in green, orange, red, black and white; respectively.

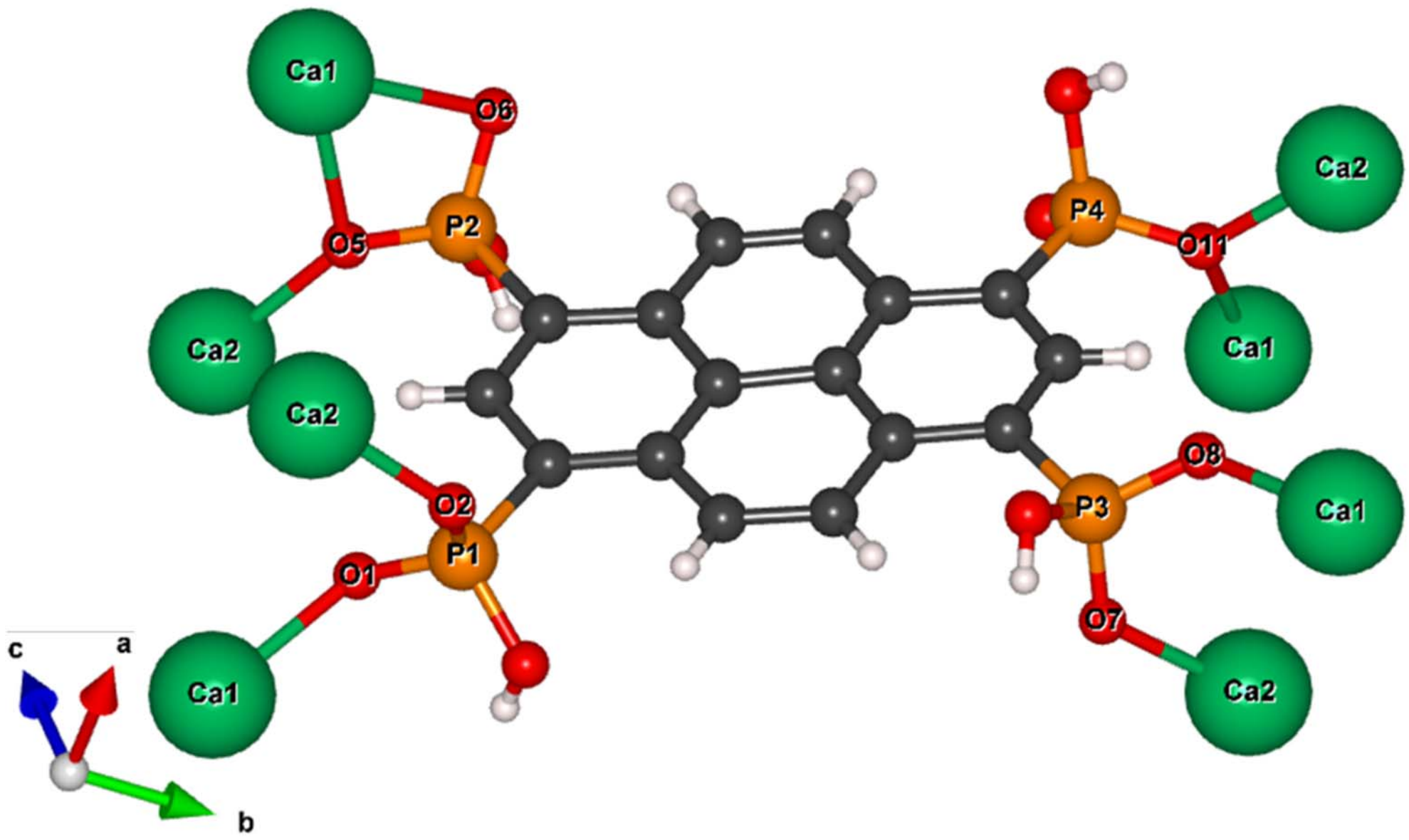

Figure S6 IEF-33 ligand connectivity. Calcium, phosphorus, oxygen, carbon and hydrogen atoms are represented in green, orange, red, black and white; respectively.

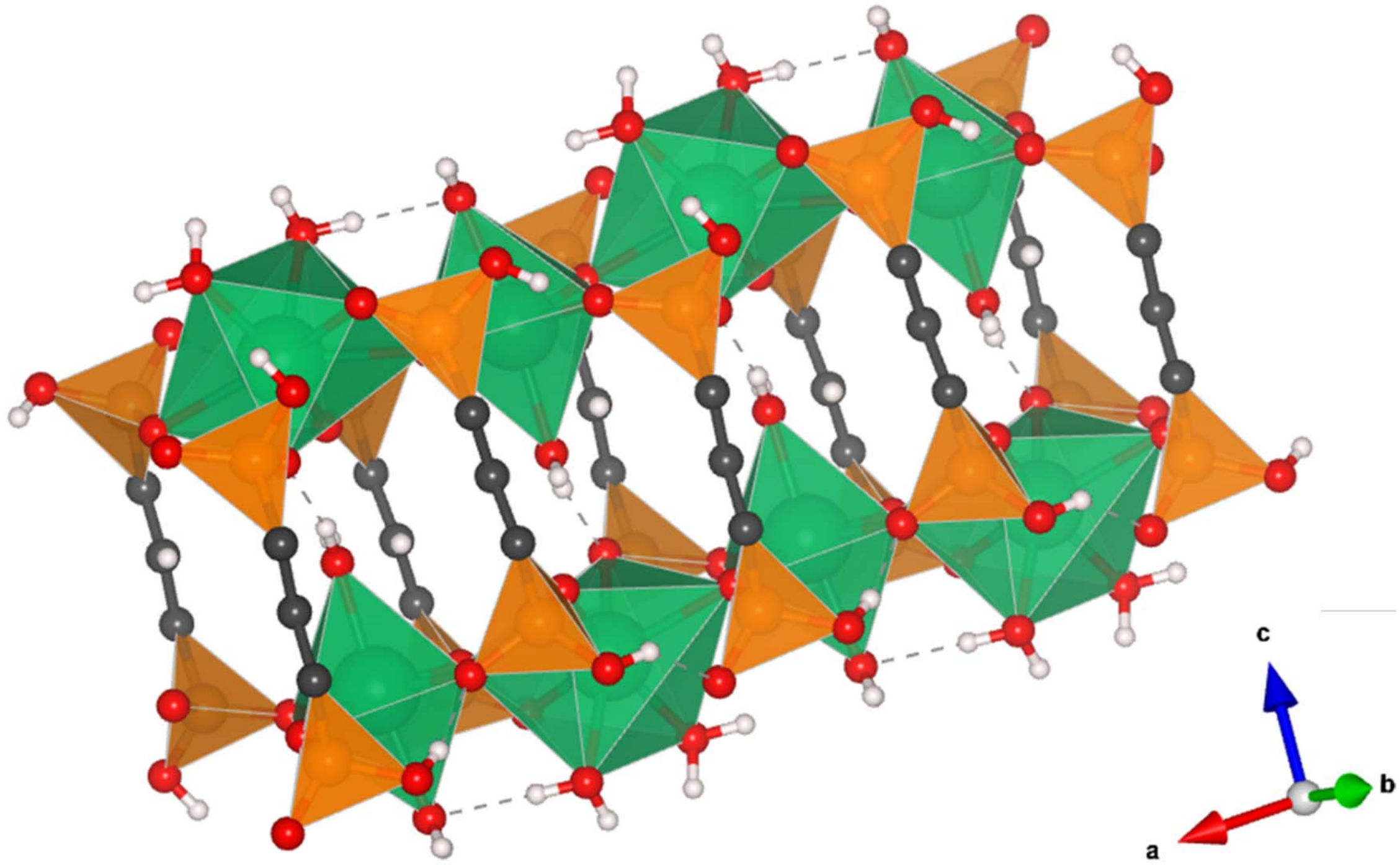

Figure S7 IEF-33 SBUs H-bond interactions. Calcium, phosphorus, oxygen, carbon and hydrogen atoms are represented in green, orange, red, black and white; respectively.

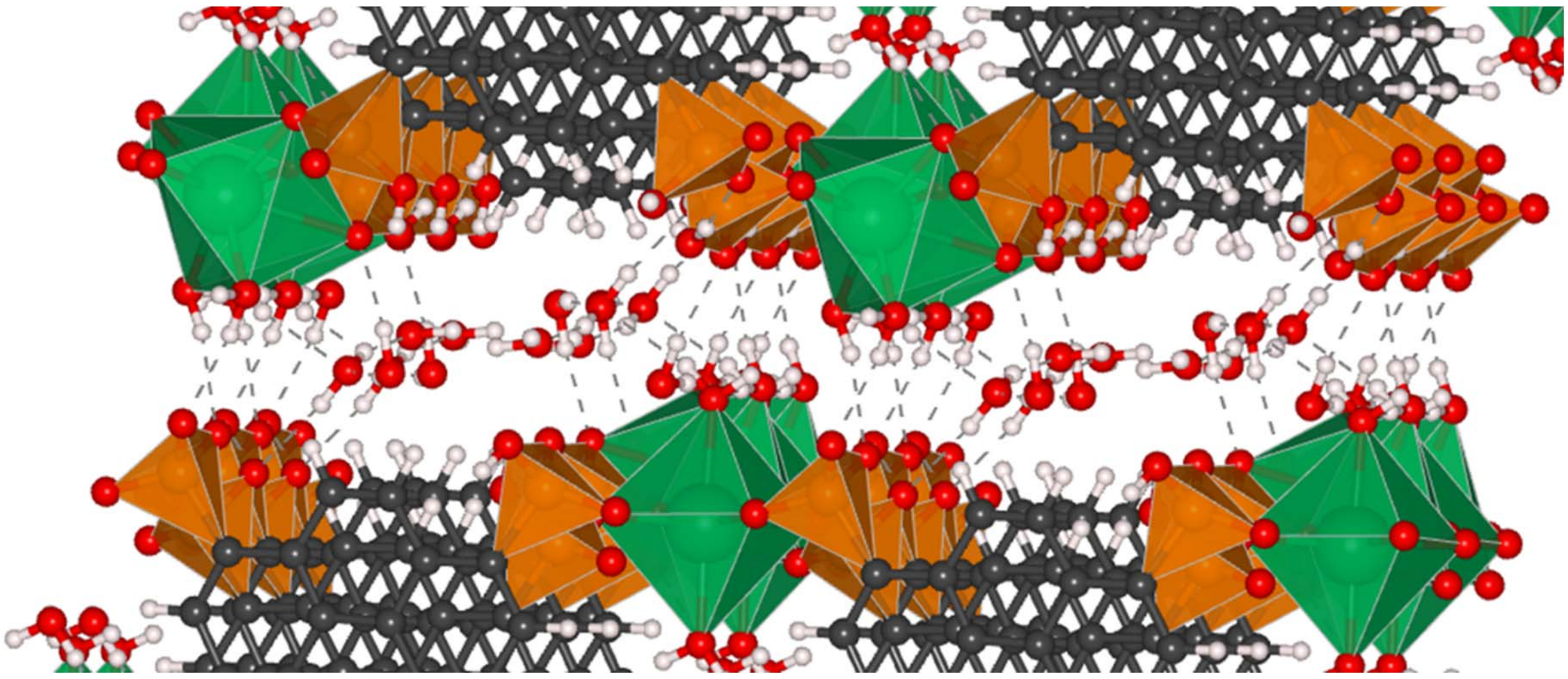

Figure S8 H-bonds interactions between IEF-33 layers and water molecules. Calcium, phosphorus, oxygen, carbon and hydrogen atoms are represented in green, orange, red, black and white; respectively.

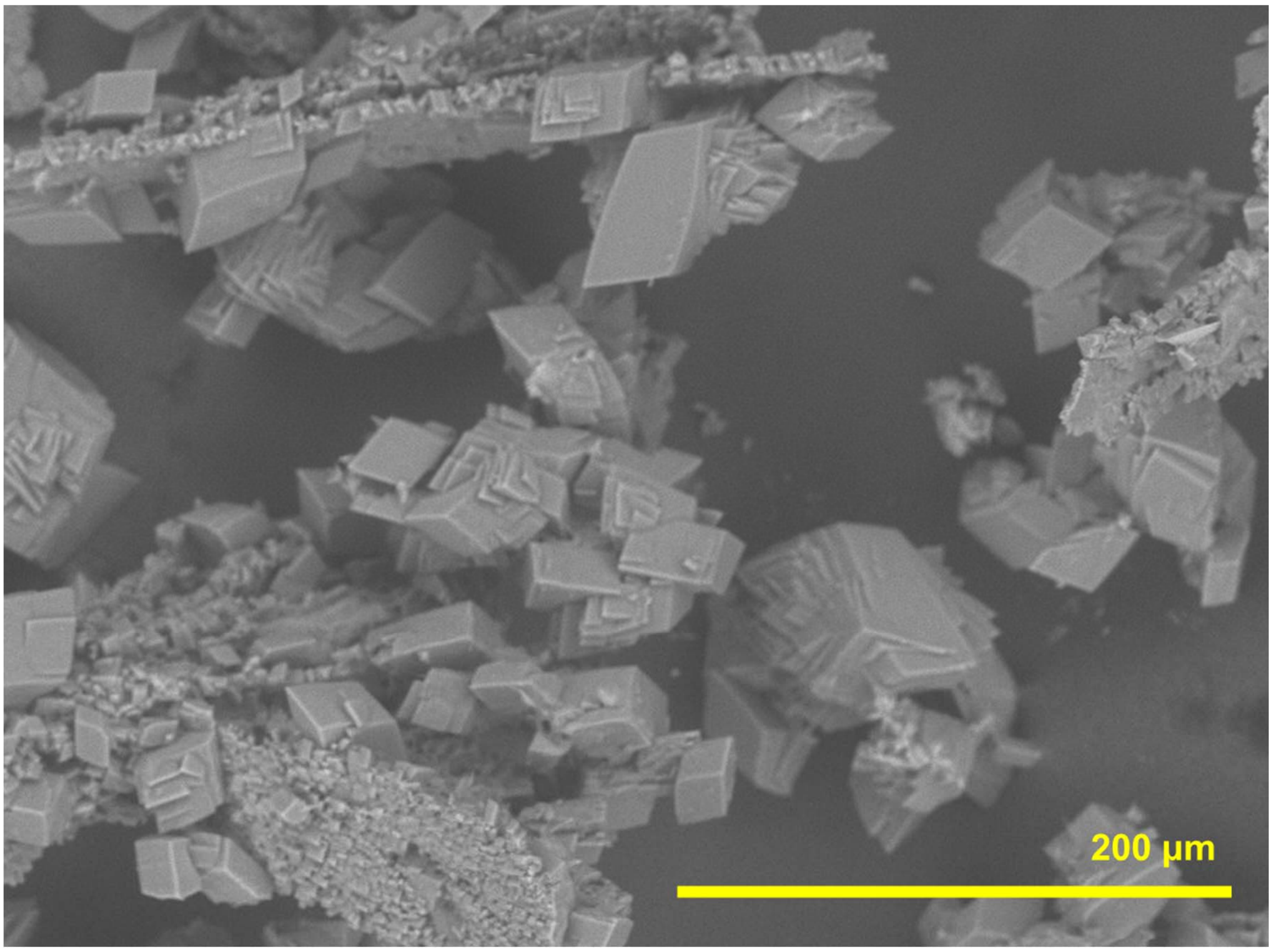

Figure S9 SEM image of IEF-34.

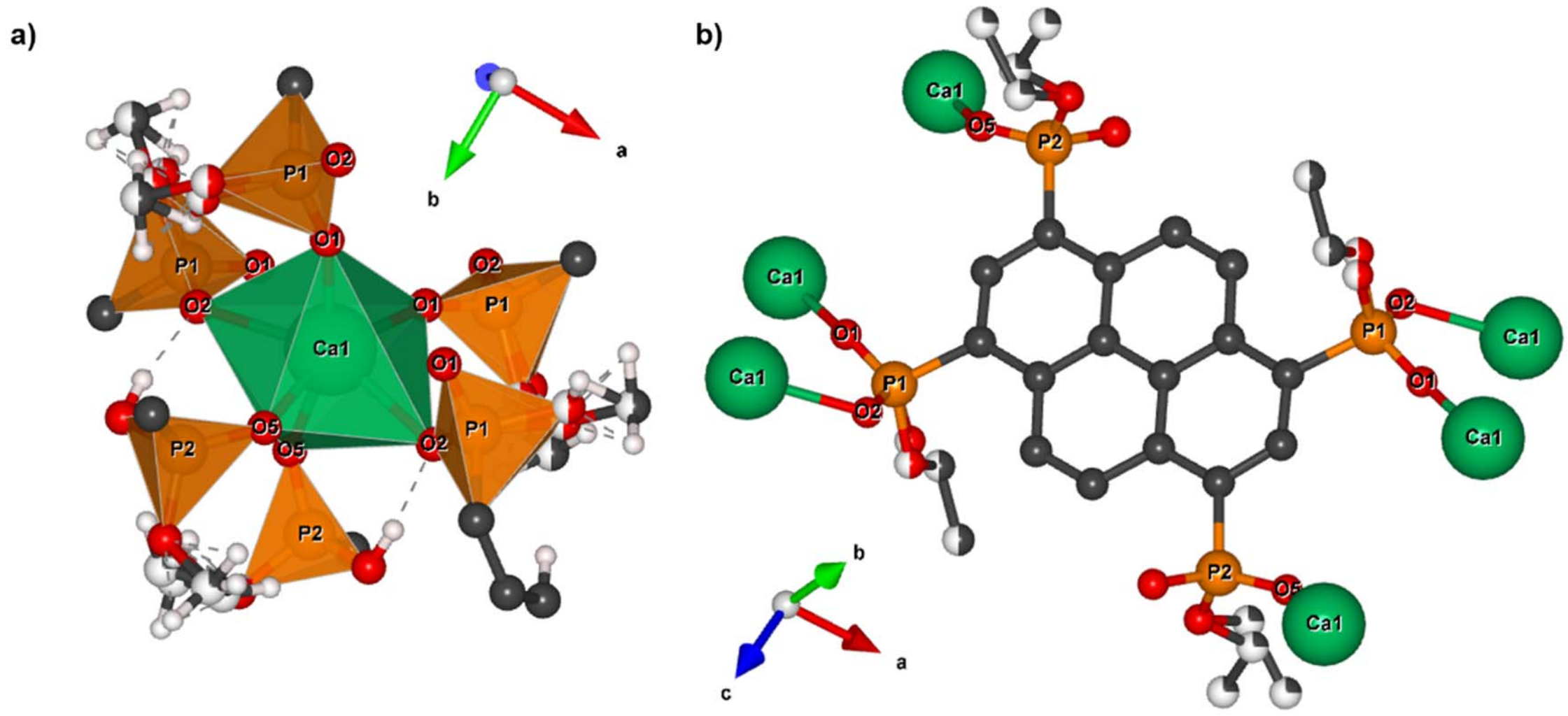

Figure S10 SBU (a) and ligand connectivity (b) of IEF-34. Calcium, phosphorus, oxygen, carbon and hydrogen atoms are represented in green, orange, red, black and white; respectively. Hydrogen atoms in (b) were removed in terms of clarity.

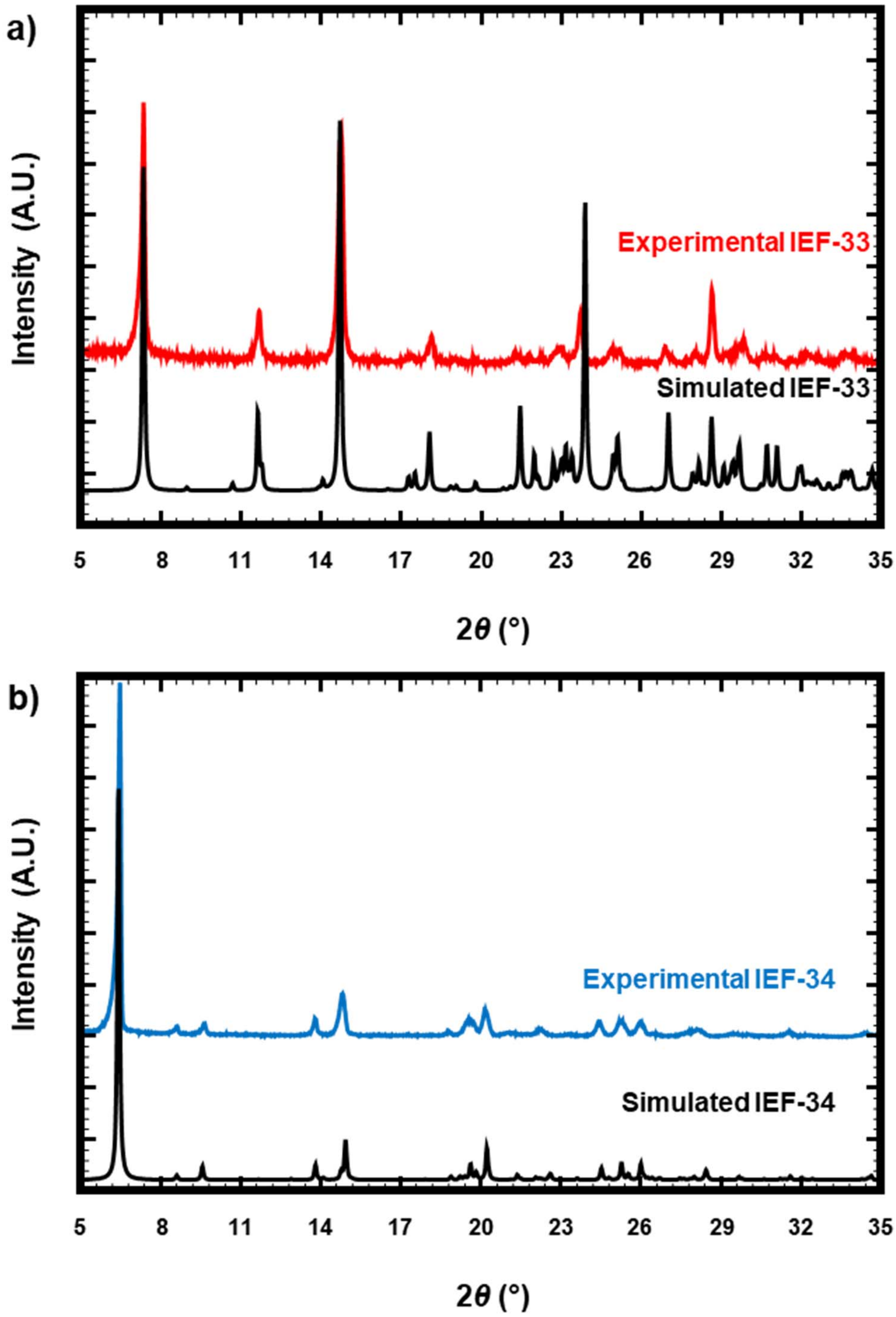

Figure S11 Comparison of PXRD patterns simulated from SCXRD data (black) and experimental (red) from IEF-33 (a) and IEF-34 (b).

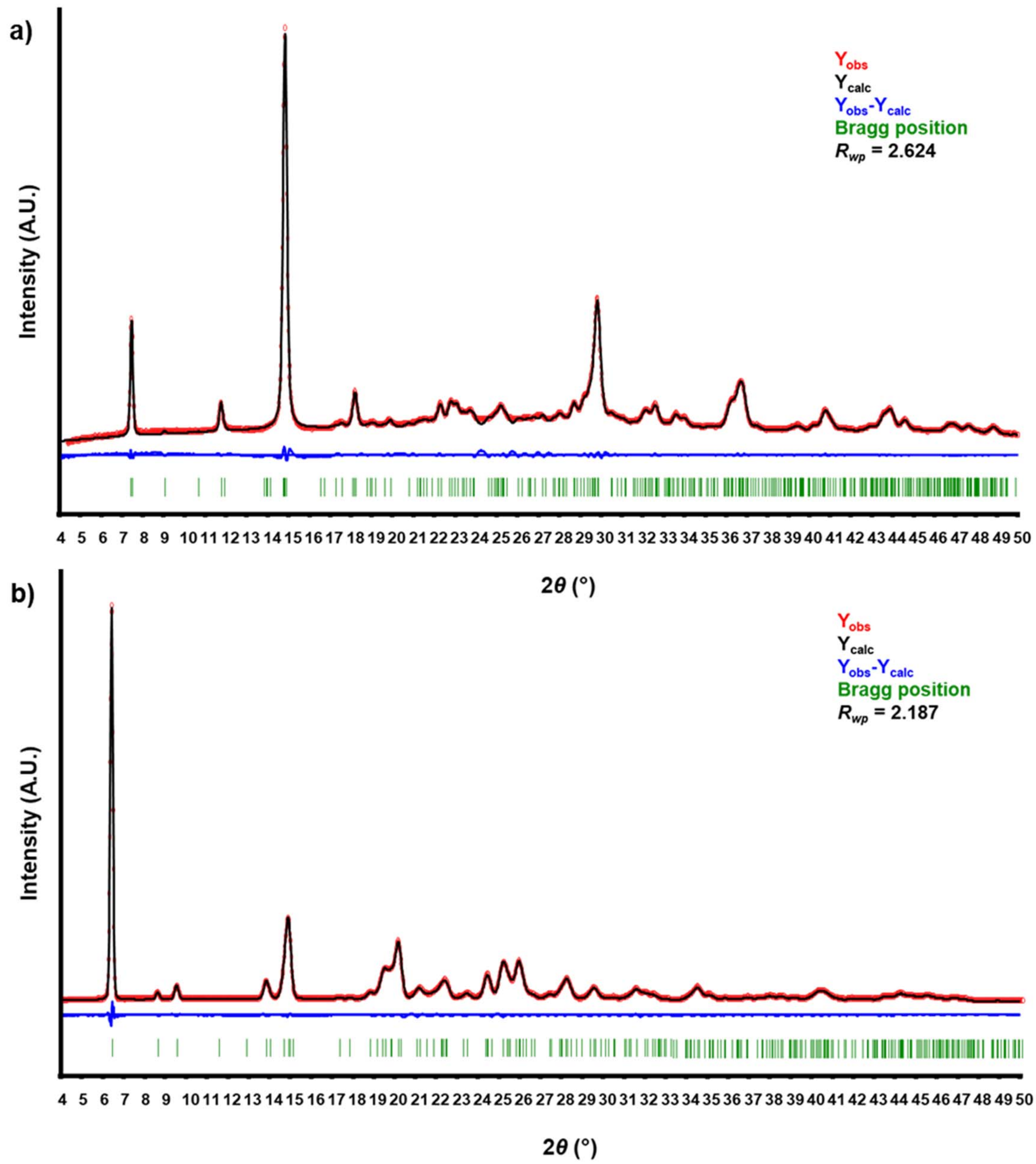

Figure S12 PXRD Pawley refinements of IEF-33 (a) and IEF-34 (b).

Table S7 Unit cell parameters of IEF-33 and IEF-34 obtained by SCXRD and PXRD.

**Material**	**IEF-33**		**IEF-34**	
Technique	*SCXRD*	*PXRD*	*SCXRD*	*PXRD*
Space group	*P*-1	*P*-1	*C*2/*c*	*C*2/*c*
a/Å	8.4993(3)	8.571(7)	21.6124(1)	21.582(3)
b/Å	12.5656(9)	12.54(1)	18.4191(8)	18.490(2)
c/Å	12.5832(2)	12.526(5)	8.3124(4)	8.400(2)
α/°	103.252(7)	103.44(7)	90	90
β/°	98.700(5)	99.04(7)	108.823(4)	108.90(2)
γ/°	97.446(4)	97.10(9)	90	90
Volume/Å^3	1274.05(2)	1275(2)	3132.0(3)	3171(1)

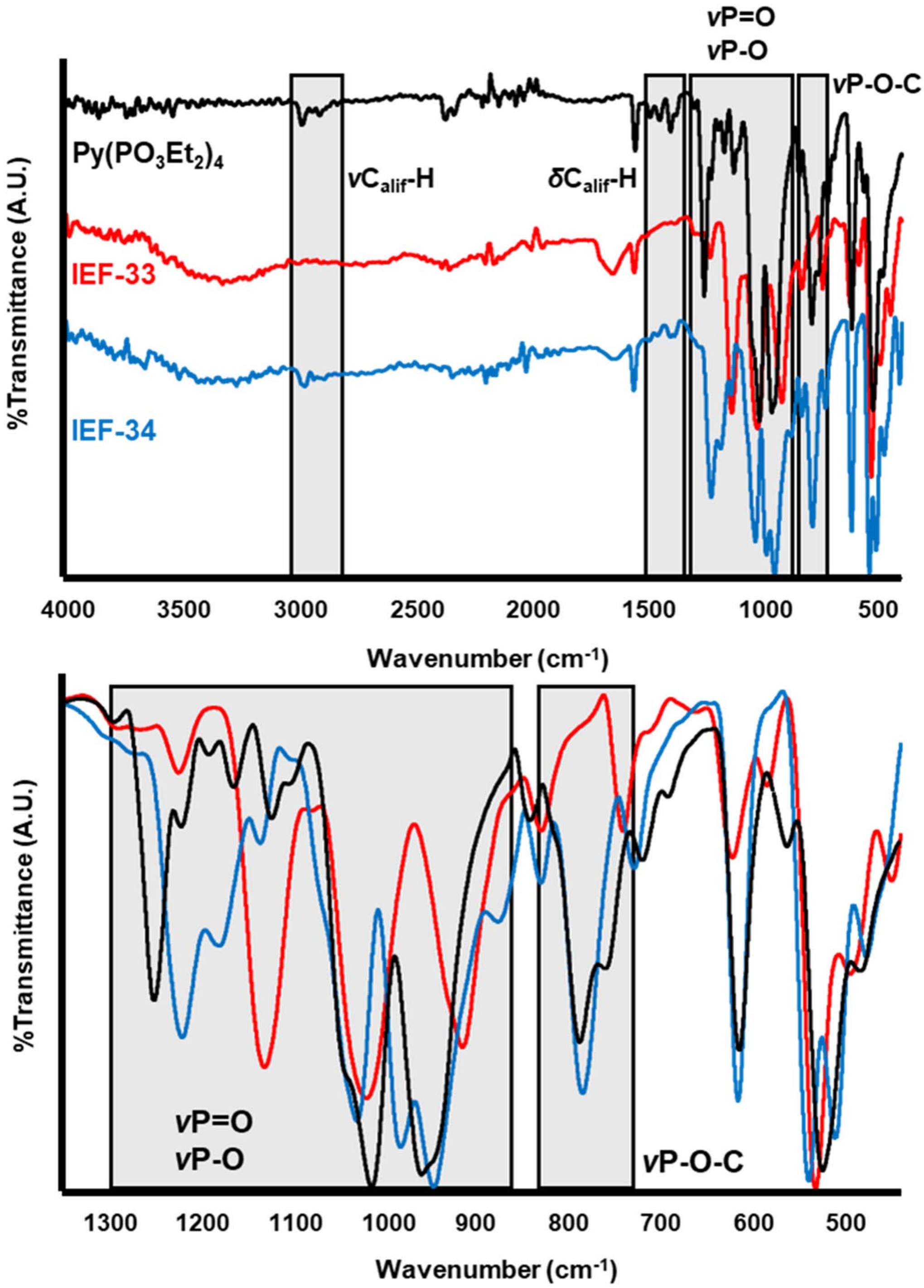

Figure S13 FTIR spectra comparison between $Py(PO_3Et_2)_4$ ligand (black), IEF-33 (red) and IEF-34 (blue).

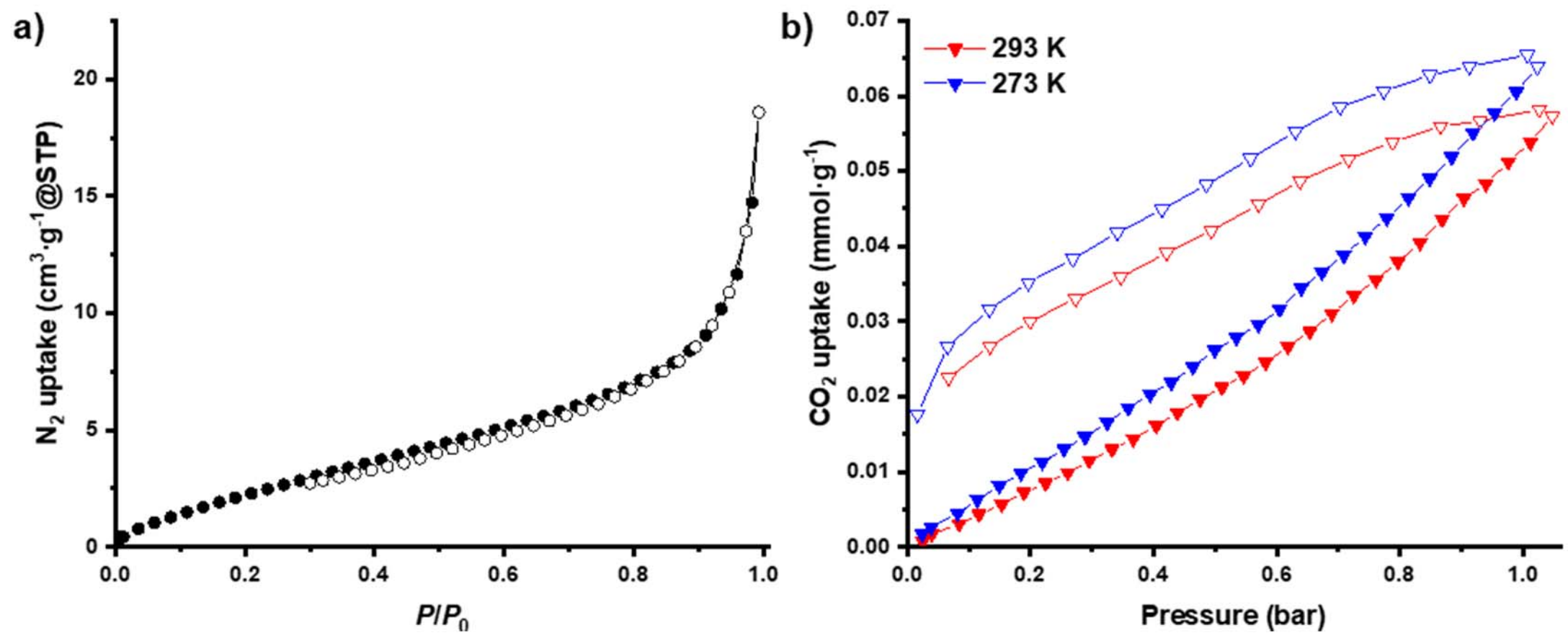

Figure S14 N_2 sorption isotherms at 77 K (a) and CO_2 sorption isotherms at 273 K (blue) and 293 K (red) (b) of IEF-33.

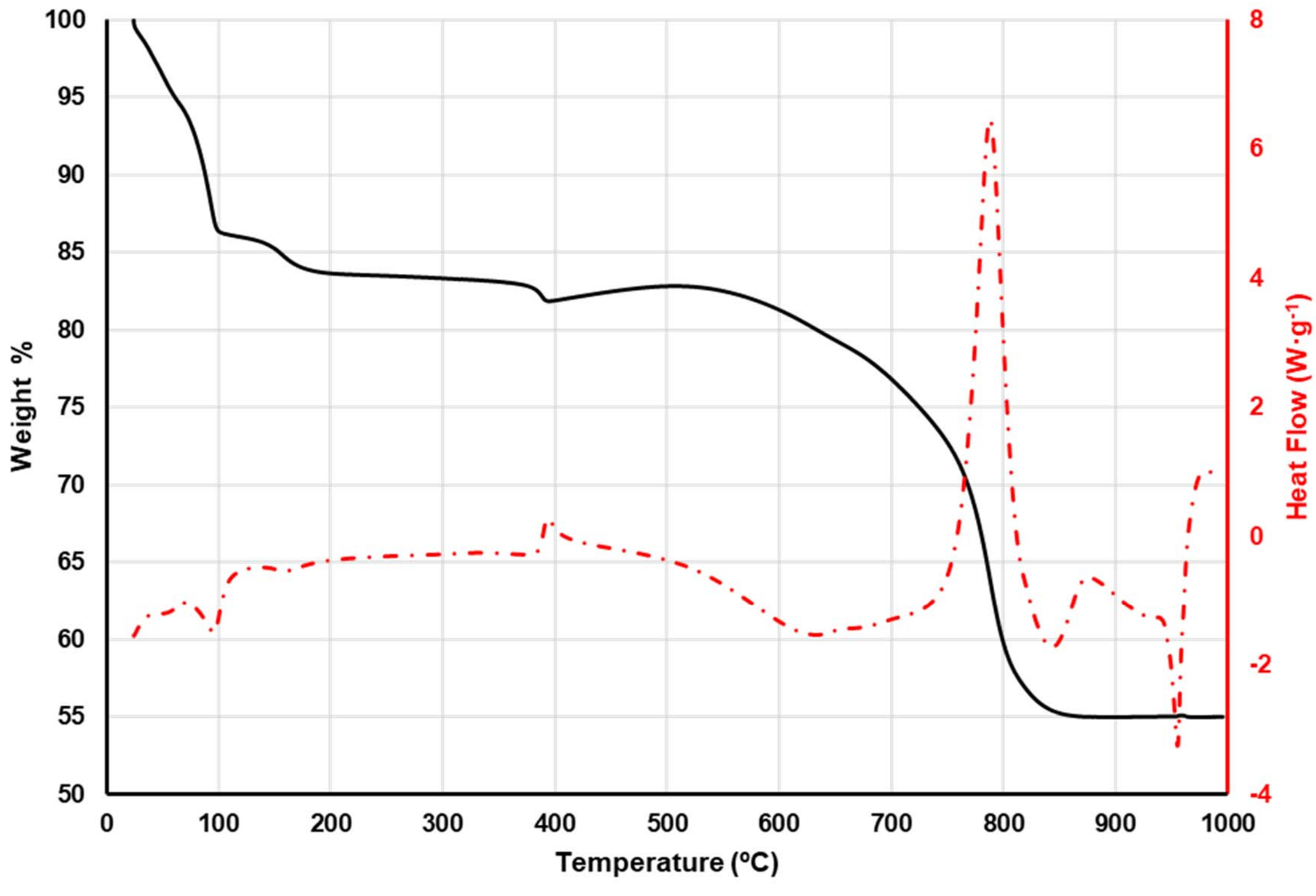

Figure S15 TGA-DSC of IEF-33 under air.

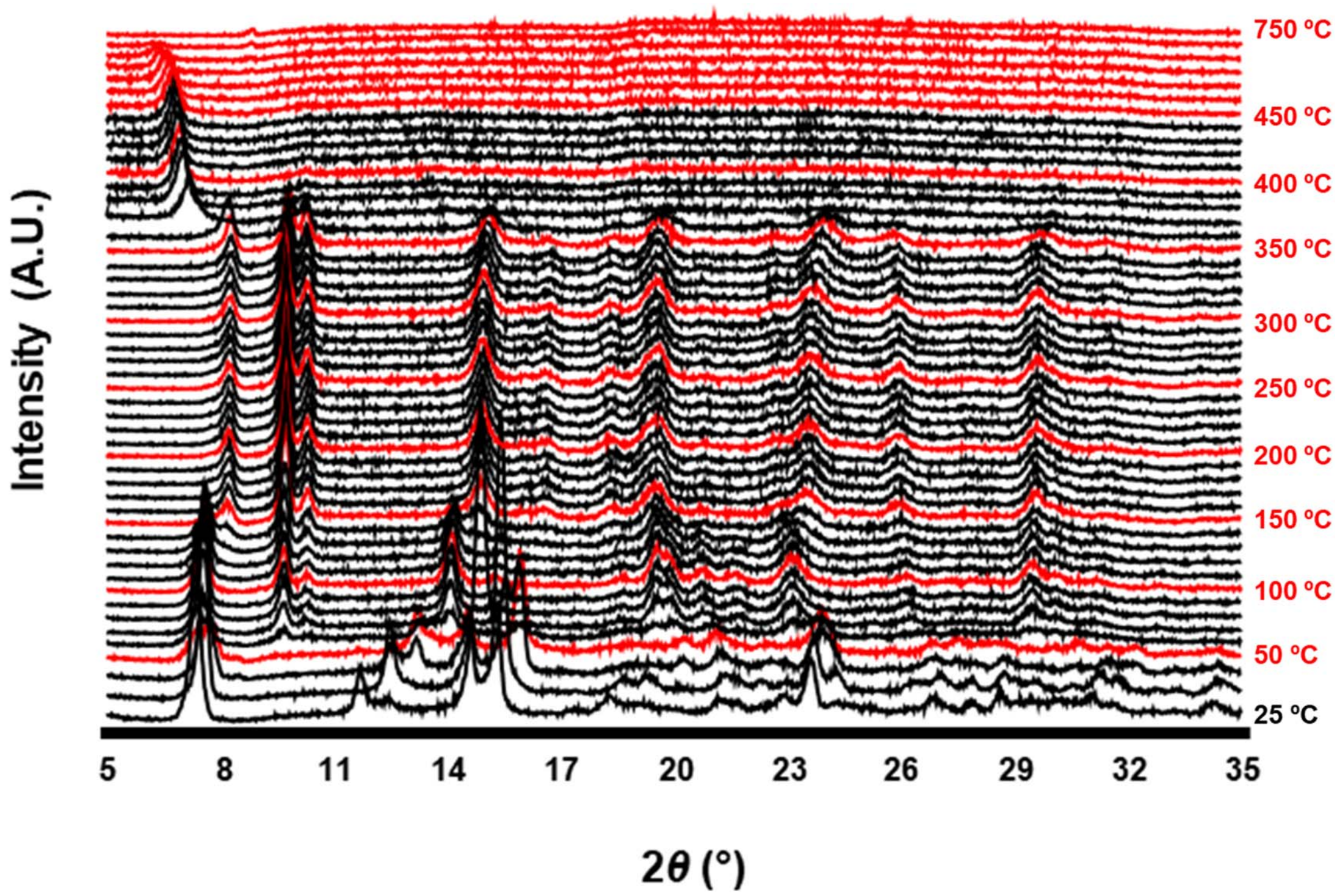

Figure S16 VTPXRD of IEF-33 under air. Each black line corresponds to a 10 ºC of increment (with the exception of the first that is 5 ºC).

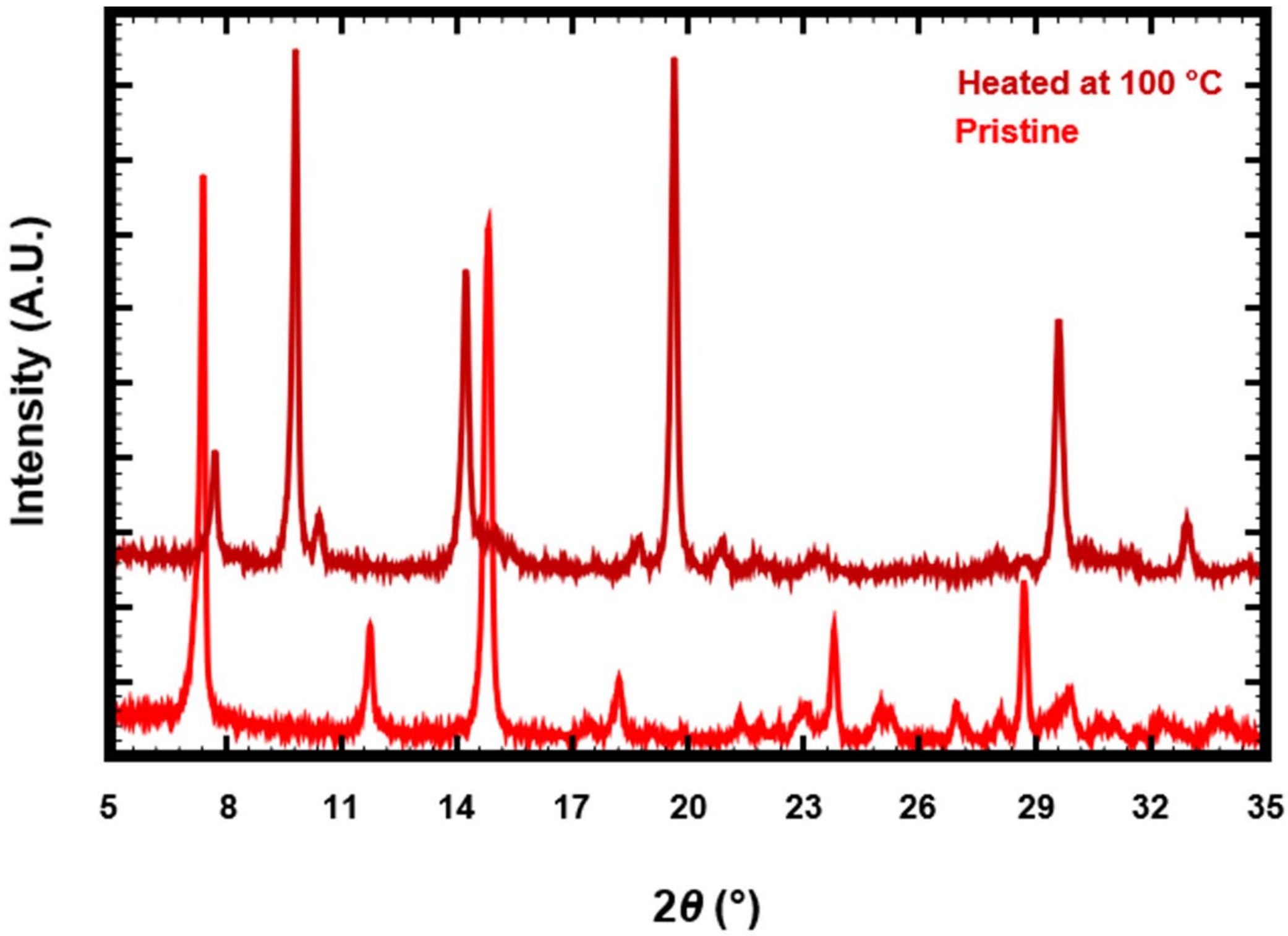

Figure S17 PXRD patterns of IEF-33 before and after heating at 100 ºC, collected at RT.

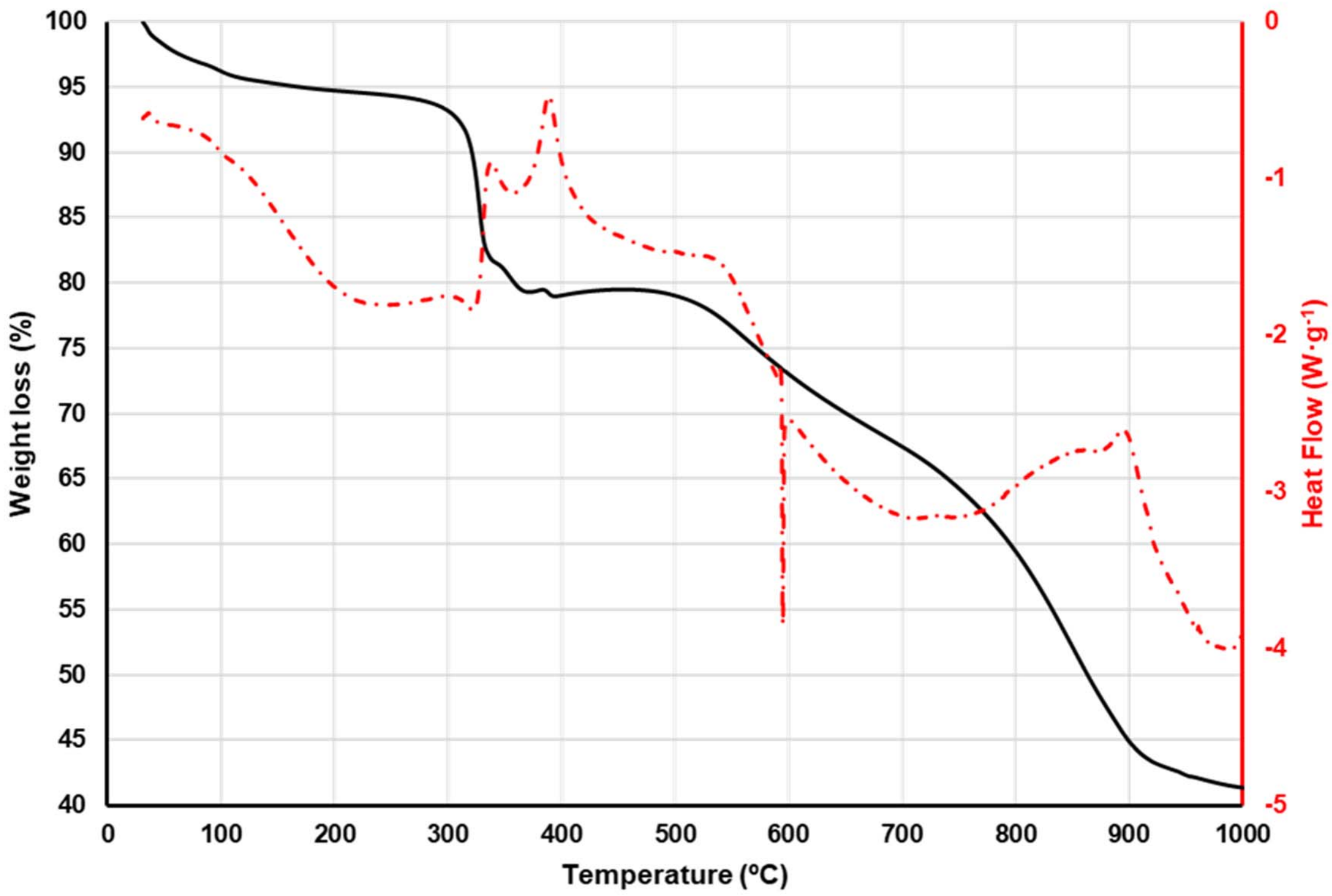

Figure S18 TGA-DSC of IEF-34 under air.

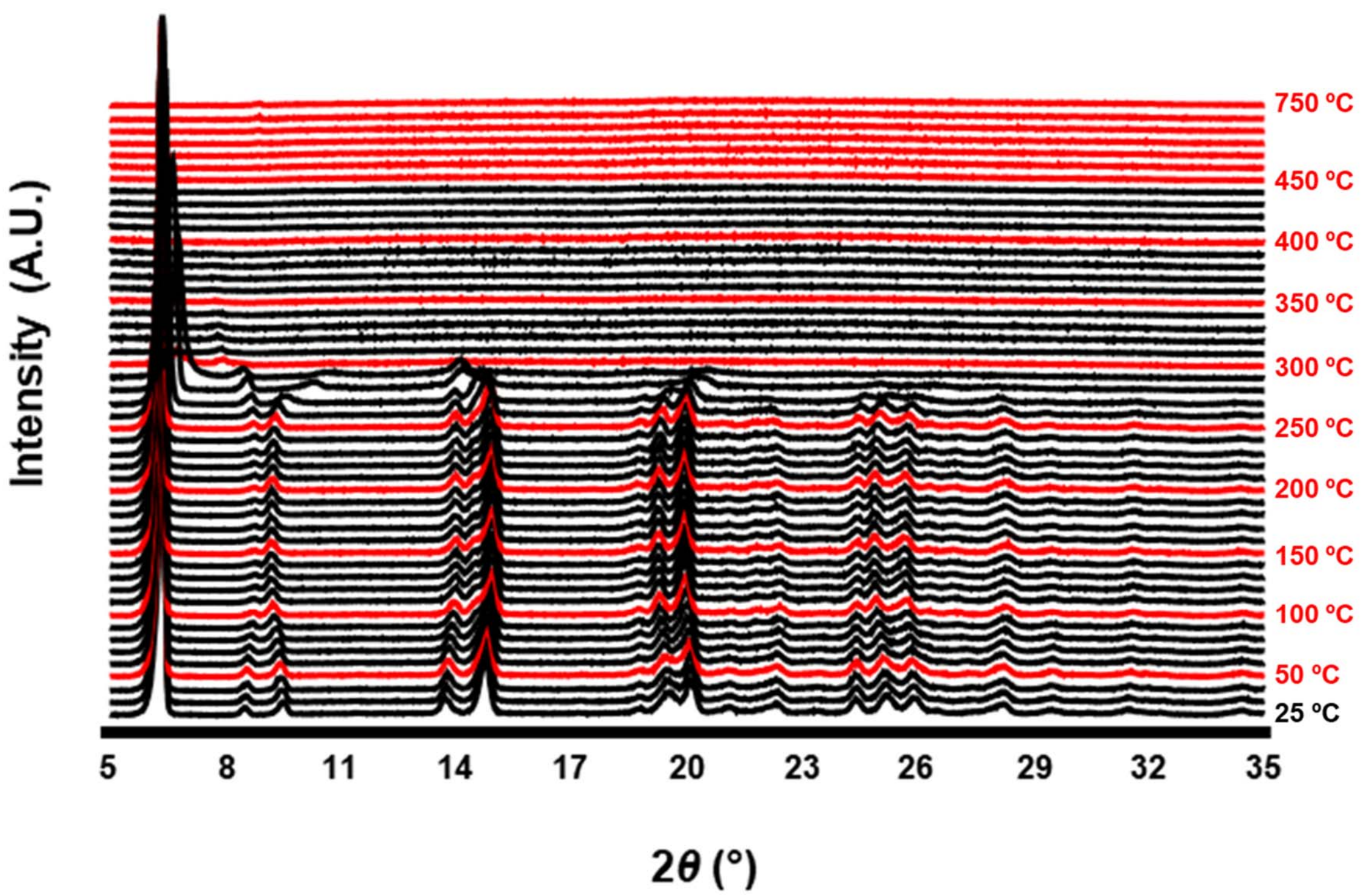

Figure S19 VTPXRD of IEF-34 under air. Each black line corresponds to a 10 ºC of increment (except for the first, that is 5 ºC).

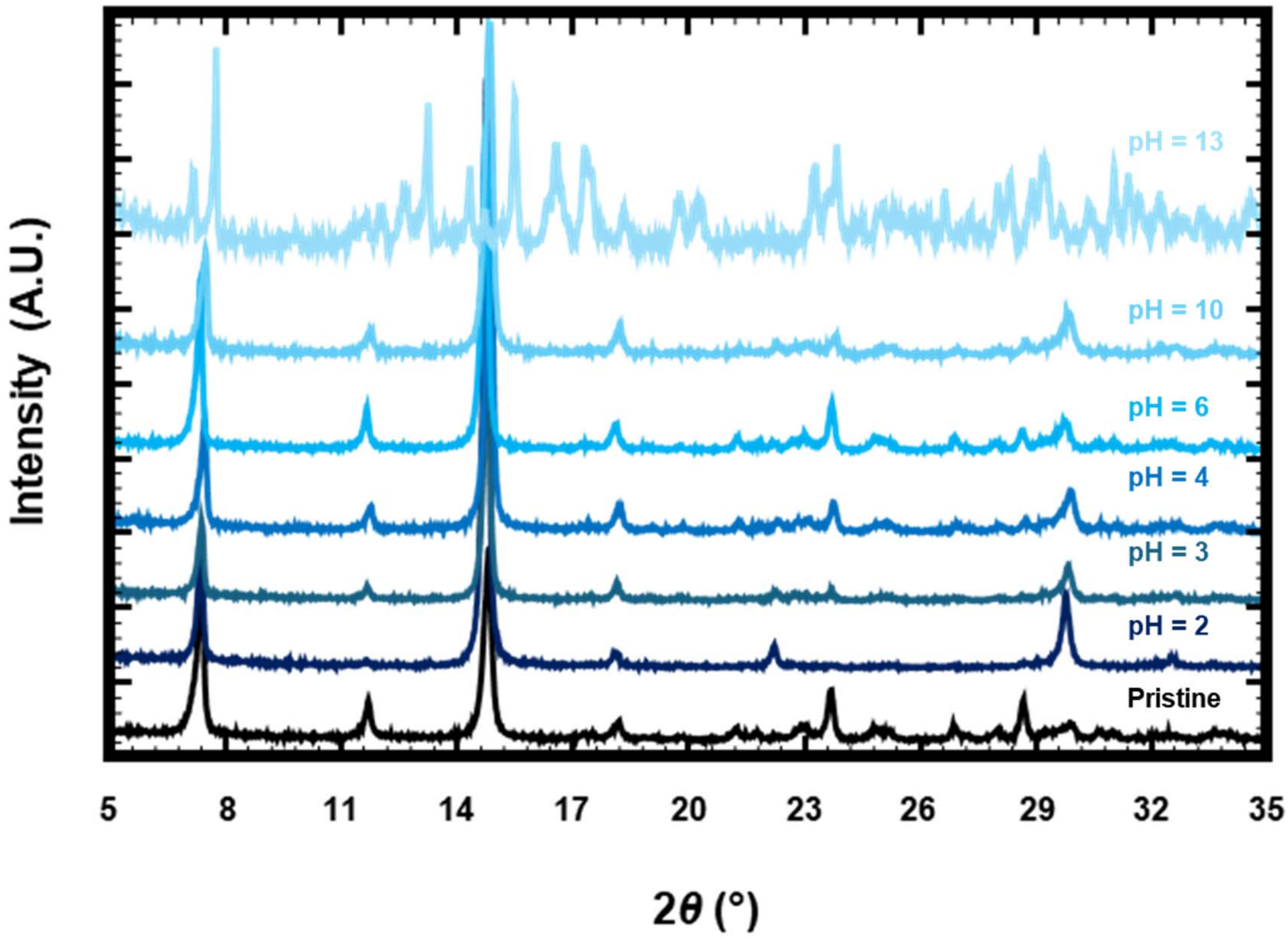

Figure S20 PXRD patterns of IEF-33 after chemical stability test in aqueous solutions at different pH.

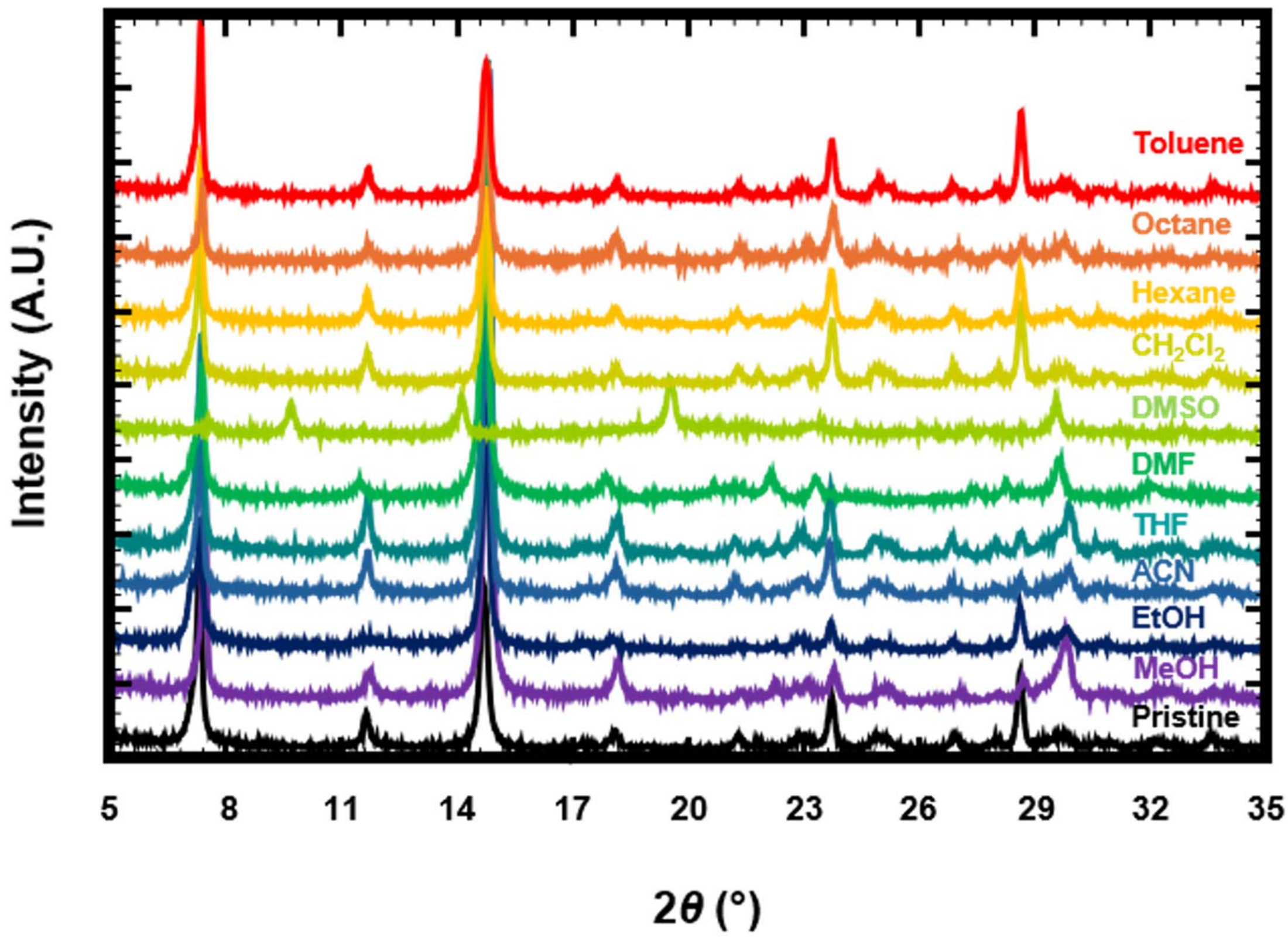

Figure S21 PXRD patterns of IEF-33 after chemical stability test in different organic solvents.

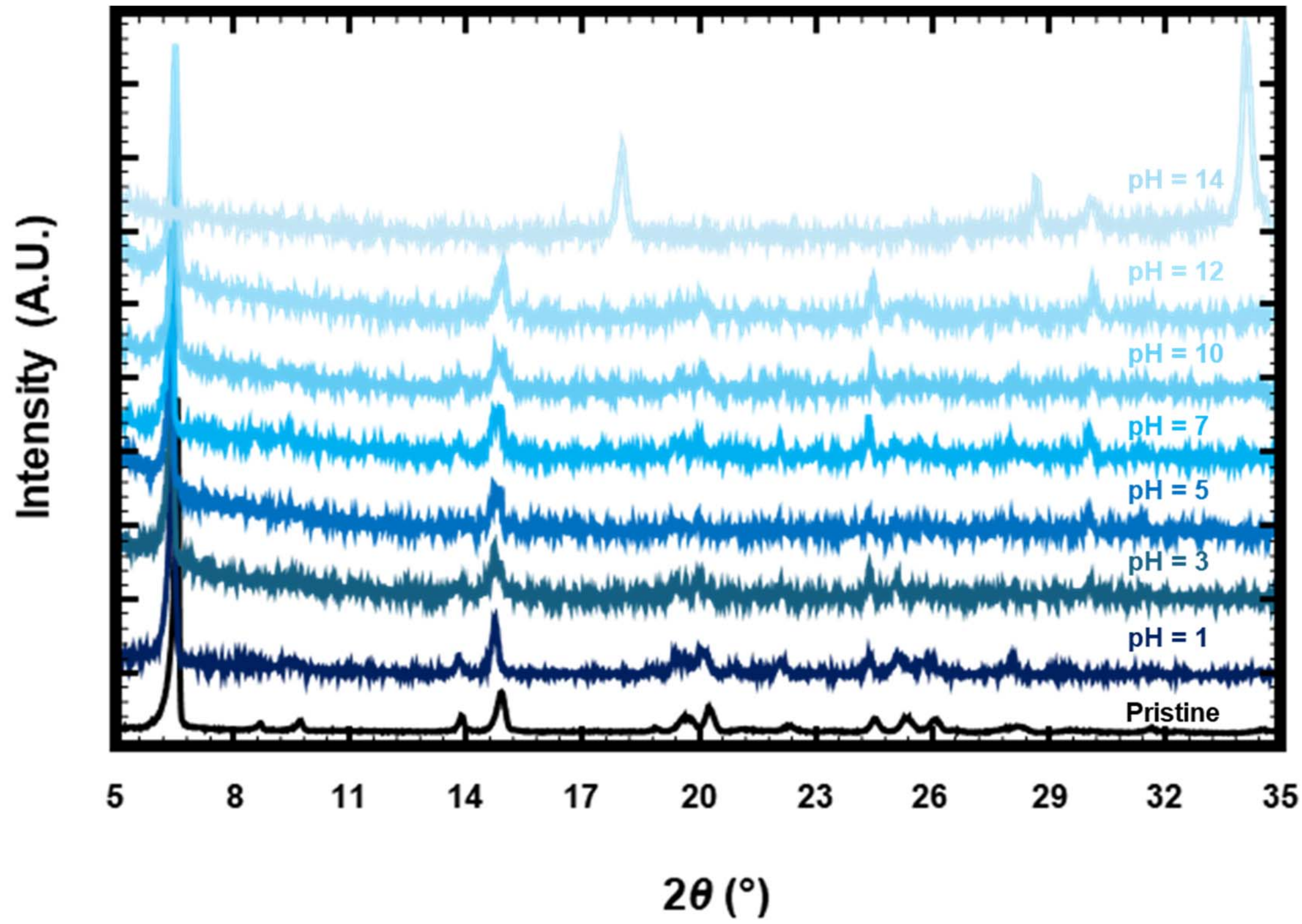

Figure S22 PXRD patterns of IEF-34 after chemical stability test in aqueous solutions at different pH.

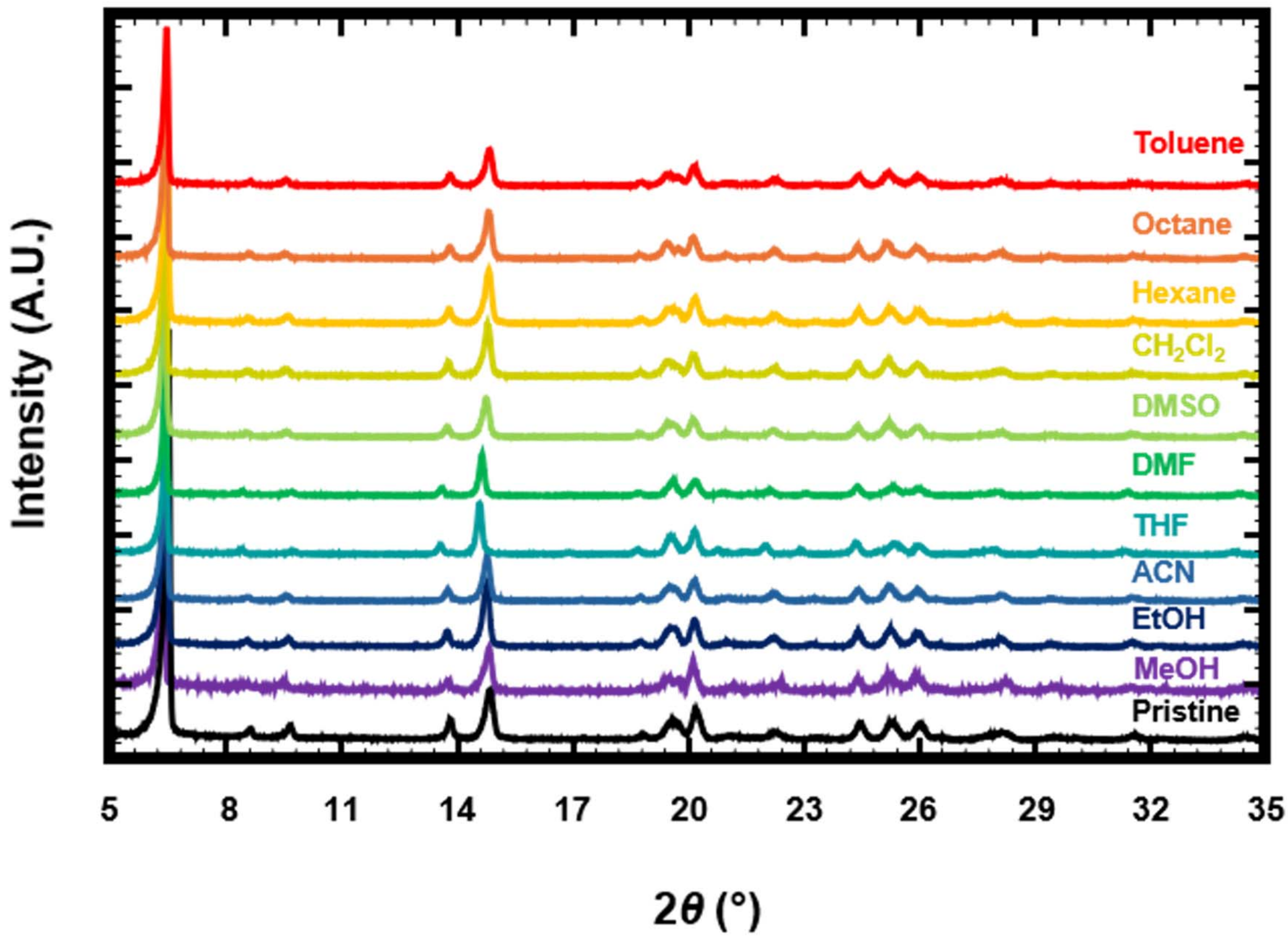

Figure S23 PXRD patterns of IEF-34 after chemical stability test in different organic solvents.

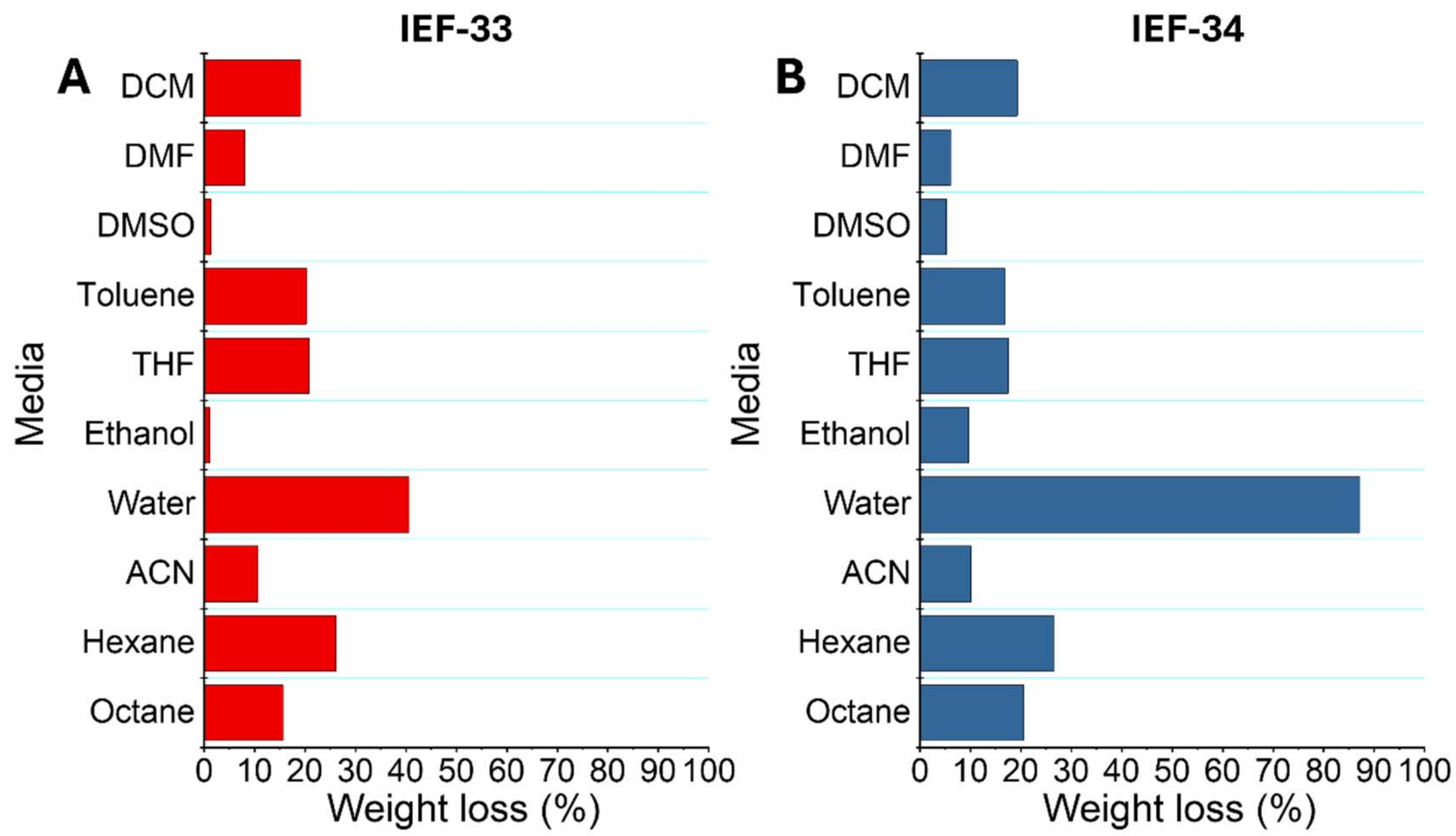

Figure S24 Gravimetric measurements after exposure to the different organic solvents and milliQ water of A) IEF-33 and B) IEF-34.

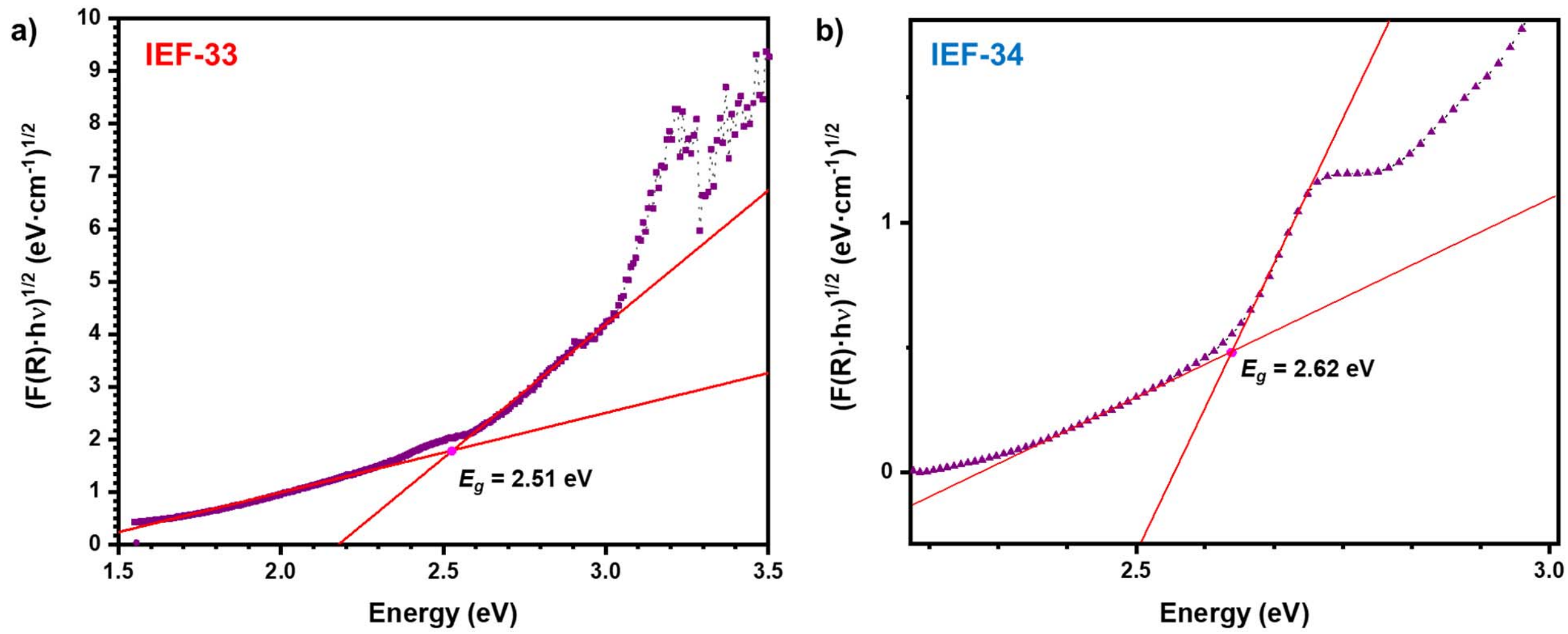

Figure S25 Tauc plot and estimated optical band gap of IEF-33 (a) and IEF-34 (b).

References

[1] S. Bernhardt, M. Kastler, V. Enkelmann, M. Baumgarten, K. Müllen, *Chem. - A Eur. J.* **2006**, *12*, 6117.
